# Double-positive T cells form heterotypic clusters with circulating tumor cells to foster cancer metastasis

**DOI:** 10.1172/JCI193521

**Published:** 2025-09-16

**Authors:** David Scholten, Lamiaa El-Shennawy, Yuzhi Jia, Youbin Zhang, Elizabeth Hyun, Carolina Reduzzi, Andrew D. Hoffmann, Hannah F. Almubarak, Fangjia Tong, Nurmaa K. Dashzeveg, Yuanfei Sun, Joshua R. Squires, Janice Lu, Leonidas C. Platanias, Clive H. Wasserfall, William J. Gradishar, Massimo Cristofanilli, Deyu Fang, Huiping Liu

**Affiliations:** 1 Department of Pharmacology,; 2 Driskill Graduate Program in Life Sciences,; 3 Division of Hematology and Oncology, Department of Medicine, and; 4 Circulating Tumor Cell Core, Robert H. Lurie Comprehensive Cancer Center, Northwestern University Feinberg School of Medicine, Chicago, Illinois, USA.; 5 Division of Hematology and Medical Oncology, Department of Medicine, Weill Cornell School of Medicine, New York, New York, USA.; 6 Department of Pathology, Immunology and Laboratory Medicine, University of Florida, Gainesville, Florida, USA.; 7 Department of Pathology, Northwestern University Feinberg School of Medicine, Chicago, Illinois, USA.; 8 Chan Zuckerberg Biohub Chicago, Chicago, Illinois, USA.

**Keywords:** Clinical Research, Immunology, Oncology, Breast cancer, Diagnostics, T cells

## Abstract

The immune ecosystem is central to maintaining effective defensive responses. However, it remains largely understudied how immune cells in the peripheral blood interact with circulating tumor cells (CTCs) in metastasis. Here, blood analysis of patients with advanced breast cancer revealed that over 75% of CTC-positive blood specimens contained heterotypic CTC clusters with CD45^+^ white blood cells (WBCs), which correlates with breast cancer subtypes, racial groups, and decreased survival. CTC-WBC clusters included overrepresented T cells and underrepresented neutrophils. Specifically, a rare subset of CD4 and CD8 double-positive T (DPT) cells was 140-fold enriched in CTC clusters versus their frequency in WBCs. DPT cells shared properties with CD4^+^ and CD8^+^ T cells but exhibited unique features of T cell exhaustion and immune suppression. Mechanistically, the integrin heterodimer α4β1, also named very late antigen 4 (VLA-4), in DPT cells and its ligand, VCAM1, in tumor cells are essential mediators of DPT-CTC clusters. Neoadjuvant administration of anti-VLA-4 neutralizing antibodies markedly blocked CTC–DPT clusters, inhibited metastasis, and extended mouse survival. These findings highlight a pivotal role of rare DPT cells in fostering cancer dissemination through CTC clustering. It lays a foundation for developing innovative biomarker-guided therapeutic strategies to prevent and target cancer metastasis.

## Abstract 2

Our findings uncover a fostering role of immunosuppressive, double-positive T cells in contact with circulating tumor cells and identify therapeutic approaches to eliminate cancer metastasis.

## Graphical abstract

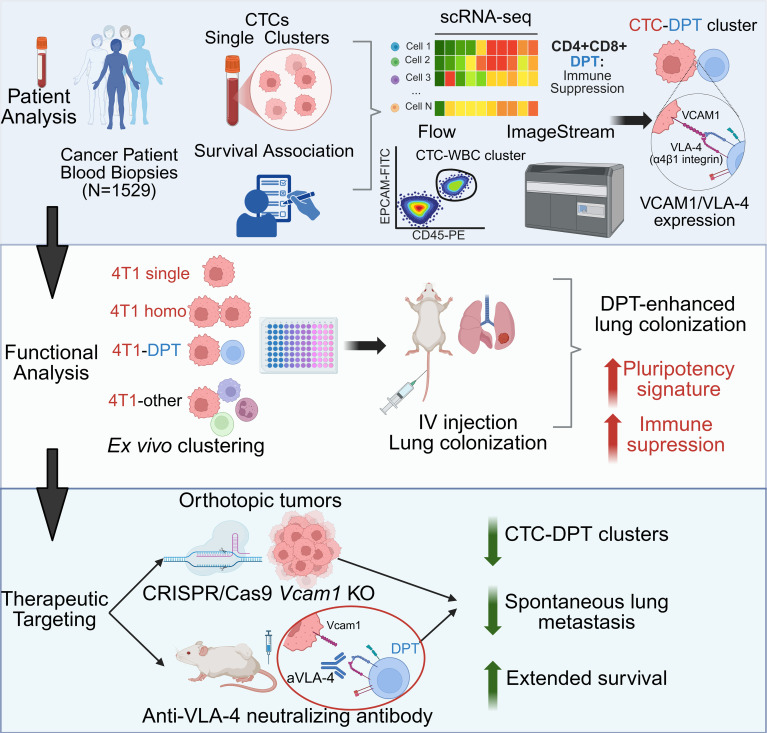

## Introduction

Circulating tumor cells (CTCs), invasive tumor cells that have entered the bloodstream with intrinsic regenerative properties and adaptive plasticity, have long been recognized as metastatic seeds of many cancers (1–13). The traditional paradigm of single-cell–mediated metastasis has been reshaped by recent discoveries that clusters of multiple tumor cells (homotypic CTC clusters) show enhanced stemness and up to 50-fold greater metastatic potential than single CTCs, correlating with worse outcomes (6, 14–21). While tumor cell fitness plays a critical role in CTC dissemination, it is pivotal — albeit technically challenging — to elucidate how immune cells, i.e., WBCs, engage with CTCs and influence metastasis.

The development and implementation of various technologies have greatly facilitated the clinical characterization of CTCs (21–25). Among these, an FDA-cleared CellSearch platform with EpCAM enrichment and immunofluorescence staining has been widely used in detecting CTCs (DAPI^+^, cytokeratin [CK^+^], and CD45^–^) from patients with epithelial cancers, such as breast (5), prostate (26), and colon (27).

Over the last 10 years, we have employed CellSearch for CTC detection in blood samples from patients with stage III and IV breast cancer (*N* = 1,529). Notably, heterotypic CTC-WBC clusters were more frequently detected than homotypic CTC clusters in CTC-positive patients, especially those with triple-negative breast cancer and luminal B subtypes, and were prognostically associated with poor survival, which is exacerbated in Black patients. We examined the cellular composition and molecular characteristics of heterotypic CTC-WBC clusters. Establishing complementary approaches of flow cytometry and ImageStream cytometry for CTC analysis (15, 17), we identified multiple immune cell types within heterotypic CTC clusters, including the relatively enriched T cells, underrepresented neutrophils, and other less frequently detected cells. Most surprisingly, a rare subset of T cells in circulation, CD4^+^ and CD8^+^ double-positive T (DPT) cells, showed the highest enrichment (140-fold) relative to WBCs, thus forming the heterotypic clusters.

DPT cells (28) are extremely rare in the periphery blood even though they are bona fide, mature T cells (29–31). To our knowledge, the role of DPT cells in cancer metastasis or CTC clusters has never been reported. Therefore, here, we determined the functional contribution of heterotypic DPT-CTC clusters to metastatic seeding and to elucidate the molecular mechanisms underlying DPT-CTC clustering.

## Results

### Clinical associations of single and clustered CTCs in breast cancer.

**Figure 1:**
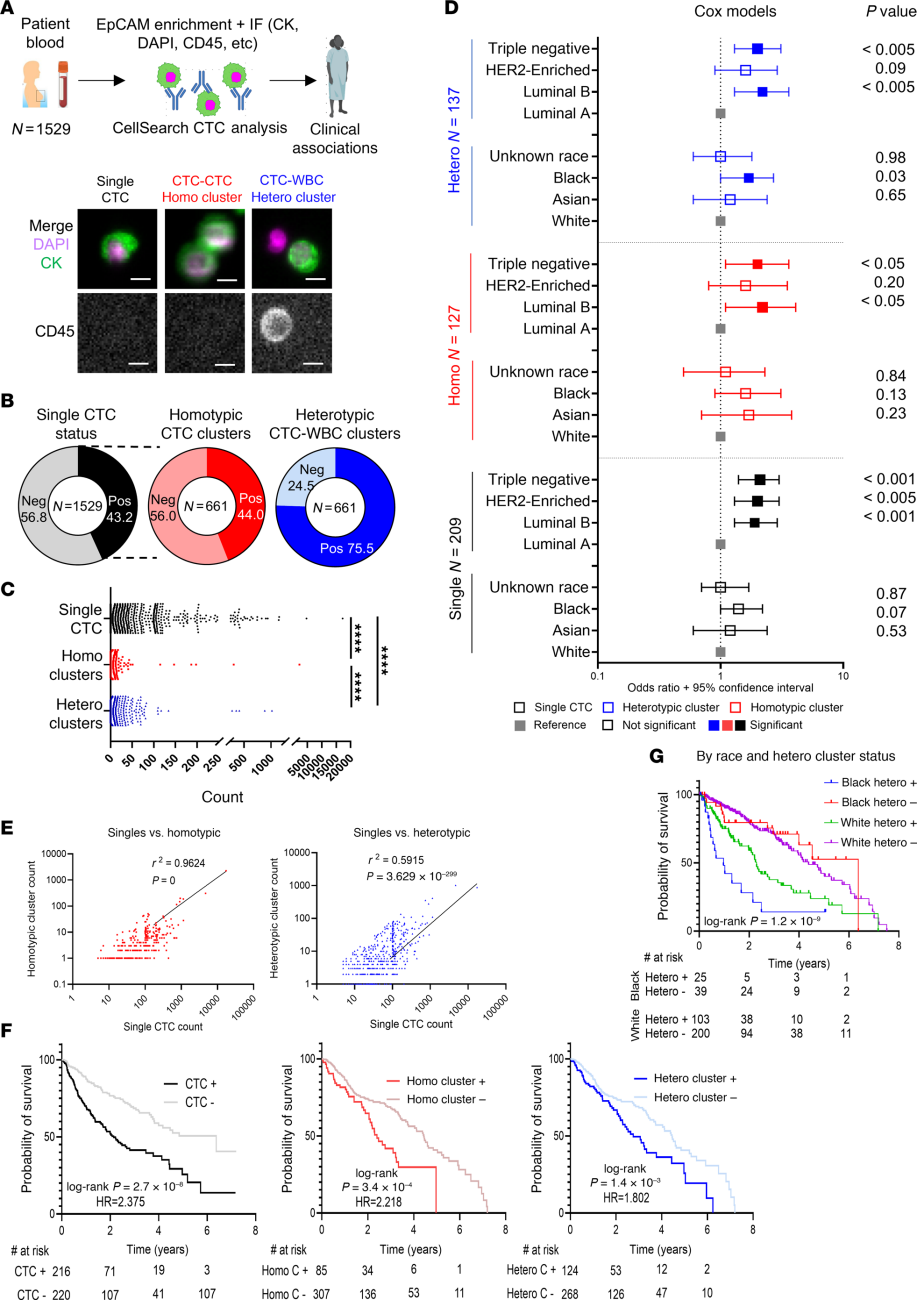
CTC frequencies in the blood biopsies of breast cancer patients and their clinical associations. (**A**) Top: Schematic of CTC analysis via CellSearch with blood specimens drawn from patients with breast cancer (*n* = 1,529). Bottom: Representative CellSearch images of single CTC (left), homotypic CTC-CTC cluster (middle), or heterotypic CTC-WBC cluster (right) with merge channels of CK (green) and DAPI (magenta) as well as a single CD45 channel. Scale bars: 10 μm. (**B**) Frequency of CellSearch-detected CTC^+^ tests/scans (≥5 CTCs within 7.5 mL blood) among breast cancer patient biopsies (left; *n* = 1,529) and frequencies of homotypic CTC-CTC clusters (middle) and heterotypic (right) CTC-WBC clusters among CTC^+^ biospecimens (*n* = 661). (**C**) Counts of single CTCs and homotypic and heterotypic CTC clusters per 7.5 mL blood in 1,529 CellSearch tests. The range, mean, and median are 0–17,427; 47; and 0 for single CTCs; 0–2,265; 3; and 0 for homotypic clusters; and 0–1,017; 6; and 2 for heterotypic clusters. ****P < 0.0001 for any 2-group comparison using Wilcoxon’s matched-pair signed rank test. (**D**) Cox proportional hazard model odds ratio plot with 95% CI for risk of single CTCs (black), homotypic CTC-CTC clusters (red), and heterotypic CTC-WBC clusters (blue) among subtypes of breast cancer and self-identified racial groups of the patients. Filled squares highlight significant features calculated using Wald’s test (*P* < 0.05). (**E**) Scatter plots of single versus homotypic clusters and single versus heterotypic clusters with Pearson’s correlation coefficient and 2-tailed *P* value. (**F**) Kaplan-Meier survival curves of patients positive for single CTCs (≥5), homotypic clusters (≥1), or heterotypic clusters (≥1) versus the patients with negative results. Log-rank (Mantel-Cox) test *P* values and hazard ratio (HR) are displayed. (**G**) Kaplan-Meier survival curves of patients with breast cancer, divided by race (Black and White) and heterotypic cluster status. Log-rank (Mantel-Cox) test *P* value is shown.

At the Northwestern University Circulating Tumor Cell Core, we established a clinical protocol of blood CTC tests for patients with stage III and IV breast cancer from 2016 to 2025 (*N* = 1,529) using the CellSearch platform coupled with image scans (Figure 1A and Supplemental Table 1; supplemental material available online with this article; https://doi.org/10.1172/JCI193521DS1). In addition to the enumeration report of single CTCs (DAPI^+^CK^+^CD45^–^), we manually reviewed the immunofluorescence images of EpCAM bead–precipitated cells for the identification of homotypic CTC clusters (≥2 CTCs/cluster) and heterotypic CTC clusters with CD45^+^DAPI^+^ WBCs (Figure 1, A and B, and Supplemental Figure 1). Of these tests, 43.23% were CTC positive (≥5 single CTCs within 7.5 mL blood). Of CTC-positive biospecimens (*N* = 661), 44.02% contained homotypic clusters (at least 1 cluster), whereas 75.49% were positive for heterotypic clusters (at least 1 cluster) (Figure 1B). The range, mean, and median counts were 0–17,427; 47; and 0 for single CTCs; 0–2,265; 3; and 0 for homotypic clusters; and 0–1,017; 6; and 2 for heterotypic clusters with a higher detection frequency of heterotypic clusters than homotypic clusters but lower than single CTCs (Figure 1C). Most heterotypic CTC-WBC clusters contained only 1 immune cell with 1 tumor cell (Supplemental Figure 1B).

We performed a Cox proportional hazard analysis to determine which patient variables would be risk factors for the detection of single CTCs, homotypic clusters, or heterotypic clusters (Figure 1D). Compared with the White group, self-identified Black or African American patients had a higher risk specifically for heterotypic clusters with no significant difference for single CTCs or homotypic clusters. When luminal A breast cancer subtype served as the reference control, luminal B, HER2-enriched, and triple-negative breast cancer (TNBC) showed higher risks of single CTCs. In contrast, only TNBC and luminal B had higher risks for homotypic and heterotypic clusters.

Single CTC counts positively correlated with both cluster types, having a stronger correlation with homotypic cluster count (*R*^2^ = 0.9624) than heterotypic cluster count (*R*^2^ = 0.5915) (Figure 1E), implying that additional factors derived from the WBCs might contribute to heterotypic cluster formation. Based on the clinical follow-up data, Kaplan-Meier analysis of these patients demonstrated what we believe to be new prognostic values of heterotypic CTC-WBC clusters and confirmed those of single CTCs (5) and homotypic CTC clusters (6, 17), all of which correlate with unfavorable survival (Figure 1F). Notably, when stratified by race and the status of heterotypic clusters, Black patients positive for heterotypic clusters had the shortest overall survival versus the other 3 groups (Figure 1G). Shortened survival when stratified by race was not observed when analyzing single CTC status, but we also observed worse overall survival in Black patients that were positive for homotypic CTC clusters, compared with White patients (Supplemental Figure 2, A–C). These data provide what we believe to be novel insights into the clinical utility of CTCs, especially heterotypic CTC-WBC clusters associated with breast cancer outcomes.

### DPT cells are enriched in heterotypic CTC-WBC clusters and promote seeding.

**Figure 2:**
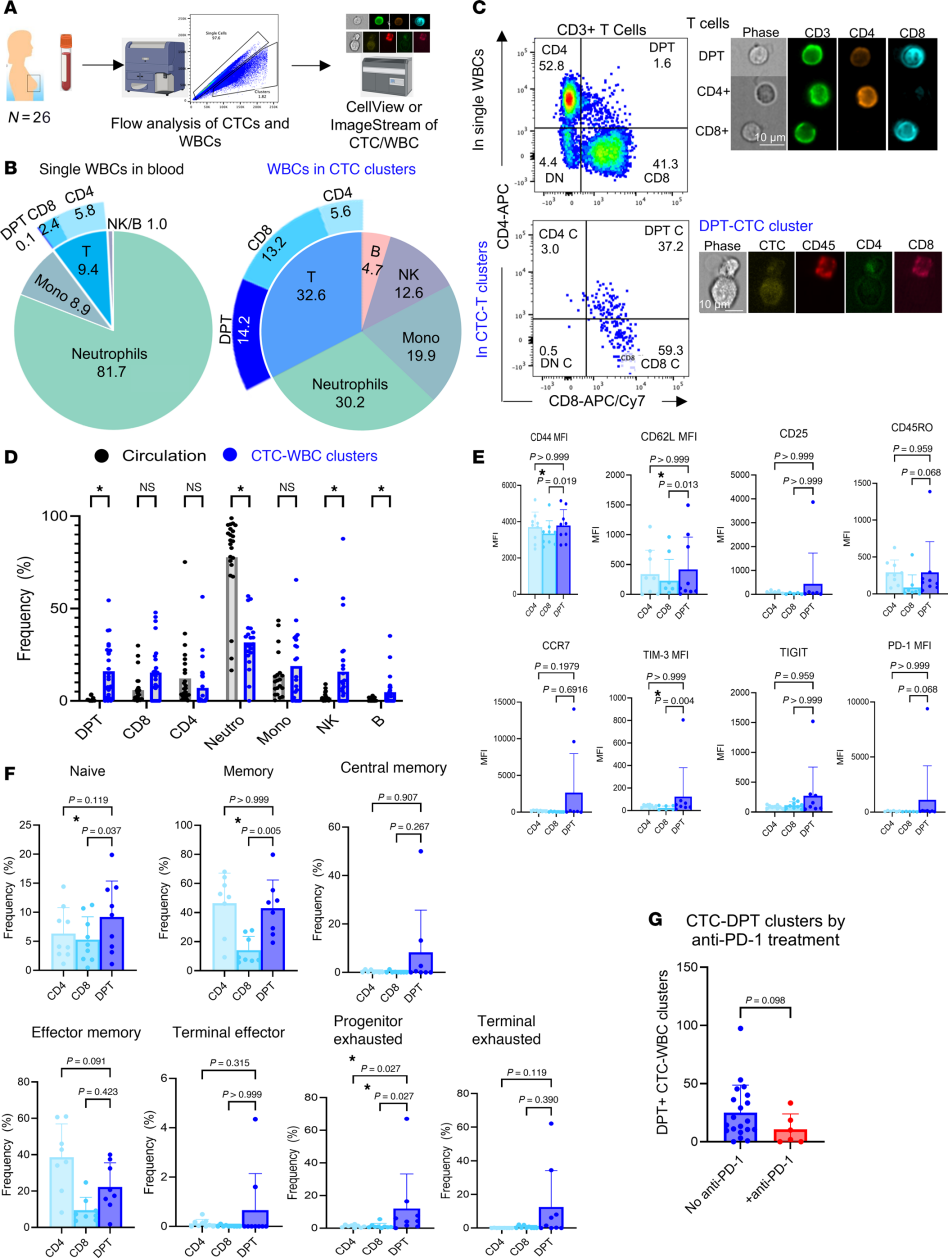
DPT cells are 140-fold enriched in CTC-WBC clusters compared with single WBCs. (**A**) Schematic of analyzing the frequency of broad classes of immune cell compositions in the blood biopsies of breast cancer patients via flow cytometry and ImageStream. *N* = 26 patients (*n* = 1,402 CTC-WBC clusters). (**B**) Frequency of immune cells (neutrophils, monocytes, NK cells, and B cells) and different T cell populations (DPT, CD4^+^ T, and CD8^+^ T) in patient blood (WBCs) (left pie chart) and CTC-WBC clusters (right pie chart). *N* = 26 patients (*n* = 698 CTC-T cell clusters). (**C**) Left: Flow dot plots of CD3^+^ T cells in single WBCs (top) and heterotypic CTC-WBC clusters (bottom). Top right: ImageStream photos of CD8^+^CD4^+^ DPT, CD4^+^T, and CD8^+^T cells. Bottom right: Representative images of CTC-DPT cluster via ImageStream imaging cytometry. Scale bars: 10 μm. (**D**) Frequency of subset T cells, neutrophils, monocytes, NK cells, and B cells from individual patients. Multiple Wilcoxon’s tests, *P < 0.05. *N* = 26. (**E**) MFI of various T cell markers (CD44, CD62L, CD45RO, CCR7, TIM-3, PD-1, CD25, and TIGIT) in human DPT cells of breast cancer patients compared with CD4^+^ and CD8^+^ single-positive T cells, as detected by flow cytometry. Friedman’s test with Dunn’s multiple-comparison test. *N* = 8. (**F**) Phenotypic characterization of human DPT cells in breast cancer patients compared with CD4^+^ and CD8^+^ single-positive T cells, including naive (CD62L^+^CD44^–^), memory (CD45RO^+^), central memory (CD45RO^+^CCR7^+^), effector memory(CD45RO^+^CCR7^–^), terminal effector (PD-1^+^TIM3^–^), progenitor-exhausted (TIM3^+^PD-1^–^), and terminal-exhausted cells (PD-1^+^TIM3^+^). *N* = 8 patients. Friedman’s test with Dunn’s multiple-comparison test. *N* = 8 breast cancer patients. *P < 0.05. (**G**) CTC-WBC clusters containing DPT cells (normalized counts) in patients who received anti–PD-1 treatment (pembrolizumab) before liquid biopsy. *N* = 20 patients who did not receive it (no anti–PD-1), and *N* = 6 patients who received it (+anti–PD-1). Mann-Whitney unpaired 2-sided *t* test.

We continued to examine the WBC composition in heterotypic clusters and their functional contribution to CTC-mediated seeding. Because CellSearch is limited to 4-channel immunofluorescence staining with only 1 channel open for customized marker analysis, we utilized established flow cytometry (15, 32) and imaging flow approaches (ImageStream and BD CellView) to characterize WBCs in heterotypic CTC clusters, based on expanded channels and cellular images (Figure 2A). After red blood cell lysis, we profiled the blood cells of 26 patients with breast cancer using extensive markers (33, 34) (plus forward and side scatter channels) to identify T cells, B cells, NK cells, monocytes, and neutrophils in single WBCs and heterotypic clusters (Supplemental Figure 3 and Supplemental Table 2). Compared with single WBCs in circulation, heterotypic CTC clusters included overrepresented T cells (32.6%), NK cells (12.6%), and B cells (4.7%), underrepresented neutrophils (30.2%), and monocytes (19.9%) without significant changes (Figure 2, B–D). The most striking identification was CD4^+^CD8^+^ DPT cells (14.2%) with a 140-fold enrichment in clusters versus a rare presence in WBCs (0.1%), in which double positivity was confirmed via ImageStream imaging cytometry (Figure 2, B–D). Additionally, the single-positive CD8^+^ and CD4^+^ T cells made up 13.2% and 5.6% of the heterotypic clusters, respectively (Figure 2, B–D).

While thymocytes go through a double-positive stage in T cell development and could become fully mature T cells (28), we found that DPT cells were relatively rare in the periphery in the blood analyses (shown above). In collaboration with the HuBMAP consortium (35, 36), we obtained spatial immunofluorescence staining images of human spleens and identified rare DPT cells close to the germinal centers (Supplemental Figure 4). We further characterized human DPT cell features in the blood biopsies of breast cancer patients using flow cytometry (Supplemental Figure 5A). DPT cells displayed a marker profile closely resembling that of CD4^+^ T cells. However, compared with CD8^+^ T cells, they had slightly higher expression levels of CD44 (activation), CD62L (trafficking; also known as L selectin), and exhaustion marker TIM3 (Figure 2E). In line with this, DPT cells had a similar distribution as CD4^+^ T cells but portrayed higher frequencies than CD8^+^ T cells in memory (CD45RO^+^) subsets (Figure 2F). Nevertheless, DPT showed higher frequencies of progenitor-exhausted phenotypes (TIM3^+^PD-1^–^) relative to single-positive T cells (Figure 2F). This suggests that DPT cells in cancer patients have an activated, exhausted, and immunosuppressive phenotype. Based on this information, we reanalyzed our flow cytometry data of CTC-DPT clusters to see if patients that received pembrolizumab (anti–PD-1 antibody) in the treatment schedule before the liquid biopsy blood sample had reduced CTC-DPT clusters. While not significant, there was a trend toward reduced CTC-DPT clusters in patients who had received pembrolizumab, suggesting that an immune checkpoint blockade may affect DPT cells and CTC-DPT clusters (Figure 2G).

**Figure 3:**
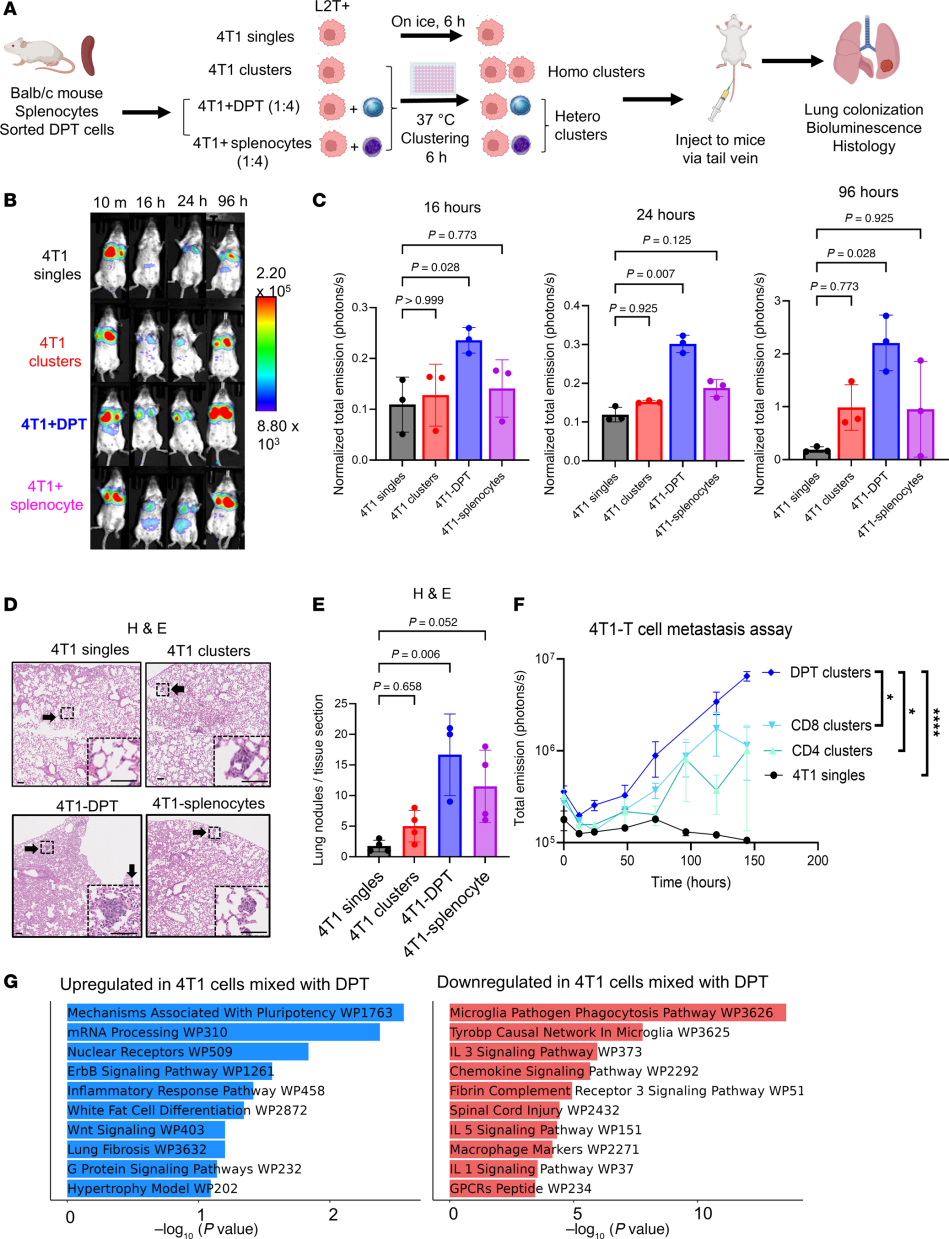
DPT tumor cell clustering promotes metastasis formation in an experimental metastasis assay in vivo. (**A**) Schematic depicting experimental design of mouse DPT isolation, clustering with L2T-labeled 4T1 tumor cells ex vivo (controls groups including 4T1 cells [singles and clusters], and 4T1 and mouse splenocytes), tail vein infusion, and lung colonization monitored via bioluminescence imaging of L2T^+^ 4T1 cells and histology validation. (**B** and **C**) Representative images (**B**) and quantification (**C**) of in vivo bioluminescent signals in mouse lungs of L2T^+^ 4T1 tumor cells after clustering and tail vein injection. Kruskal-Wallis test with multiple comparison; *n* = 3 mice per group. (**D** and **E**) H&E staining images with inserted regions of control or micrometastasis in the mouse lungs (**D**) and quantification of metastasis lesions of lung sections (**E**) on day 6 after infusion of 4 groups of cells: 4T1 singles, 4T1 homoclusters, 4T1-DPT clusters, and 4T1-splenocytes. Arrows point to metastatic lesions. Scale bars: 100 μm. Three-way ANOVA with Dunn’s multiple-comparison test was used for *P* value calculations. *N* = 3 for 4T1-DPT and 4 for all other groups. (**F**) Repeated experiment of DPT-4T1 clustering–promoted metastatic seeding and colonization with single CD4^+^ and CD8^+^ T cell controls in mix clustering with 4T1 cells. Data in graphs represent mean ± SEM. Unpaired 2-tailed *t* test; *P < 0.05, ****P < 0.001. *N* = 6 biological replicates. (**G**) Enriched pathways upregulated (left) or downregulated (right) in 4T1 cells after being incubated with DPT cells versus those with splenocytes for 6 hours, identified via the Enrichr database (https://maayanlab.cloud/Enrichr/) (WikiPathways [WP]) based on scRNA-Seq (10X Genomics) data. The respective UMAP plots for each group are provided in Supplemental Figure 6B.

Next, we sought to determine the role of DPT cells in heterotypic cluster-mediated metastatic seeding during experimental lung colonization in syngeneic mouse models (Figure 3A). After splenocytes were harvested from BALB/c mice, DPT cells were isolated via high-speed sorting (see Methods). These cells were premixed at 4:1 ratio with murine TNBC 4T1 cells (Luc2-tdTomato/L2T labeled) (37) and seeded in a poly(2-hydroxyethyl methacrylate)–coated (poly-HEMA–coated) 96-well plate for 6-hour cluster formation ex vivo (Supplemental Figure 6A). Mixed cells and clusters were collected gently and immediately infused into the tail veins of BALB/c recipient mice, and mice were immediately imaged using bioluminescent imaging. Compared with 3 control groups of 4T1 singles, 4T1 mixture with homotypic clusters, and 4T1-splenocyte mixture, 4T1-DPT mixture seeded to the lungs with the highest efficiency and strongest bioluminescence signal at 16, 24, and 96 hours (Figure 3, B and C). H&E staining of the mouse lungs 6 days after injection validated the better colonization by 4T1-DPT clusters compared with other groups (Figure 3, D and E).

As single-positive T cells were observed to be clustered with CTCs in patient blood, we also compared the seeding efficiency of the DPT-4T1 mixture with CD4-4T1 and CD8-4T1 mixtures. Similarly, infusion of DPT-4T1 cells resulted in the highest lung bioluminescent signal of disseminated tumor cells 6 days after tail vein injection compared with these 2 groups and other controls (Figure 3F and Supplemental Figure 7). These data demonstrate that DPT cells of heterotypic clusters promote CTC-mediated pulmonary seeding and colonization.

To elucidate DPT interaction–mediated effects on tumors, we conducted single-cell RNA-Seq (scRNA-Seq) of 4T1 tumor cells incubated with sorted DPTs or splenocytes for 6 hours of clustering. Tumor cells and immune cells from each condition (DPT or splenocytes) were collected together, stained with multiplexing hashtag antibodies for downstream identification, analyzed using scRNA-Seq, and plotted using Uniform Manifold Approximation and Projection (UMAP). The 4T1 tumor cells that were clustered ex vivo with either DPT cells or splenocytes did not separate strongly from each other on the UMAP; however, differentially expressed genes were identified between the tumor cells in these 2 groups (Supplemental Figure 6B and Supplemental Table 3). Using the pathway analysis tool Enrichr (38), we identified DPT-upregulated pathways in tumor cells (pluripotency, mRNA processing, nuclear receptors, and ErbB signaling) as well as markedly downregulated pathways in immune activation–related phagocytosis; IL-3, IL-5, and IL-1 signaling; and chemokine signaling (Figure 3G and Supplemental Table 4). These data suggest that the DPT interaction promotes stemness-related pluripotency and immune evasion of tumor cells.

### ITGB1 and ITGA4 are drivers of heterotypic T cell clustering with tumor cells.

**Figure 4:**
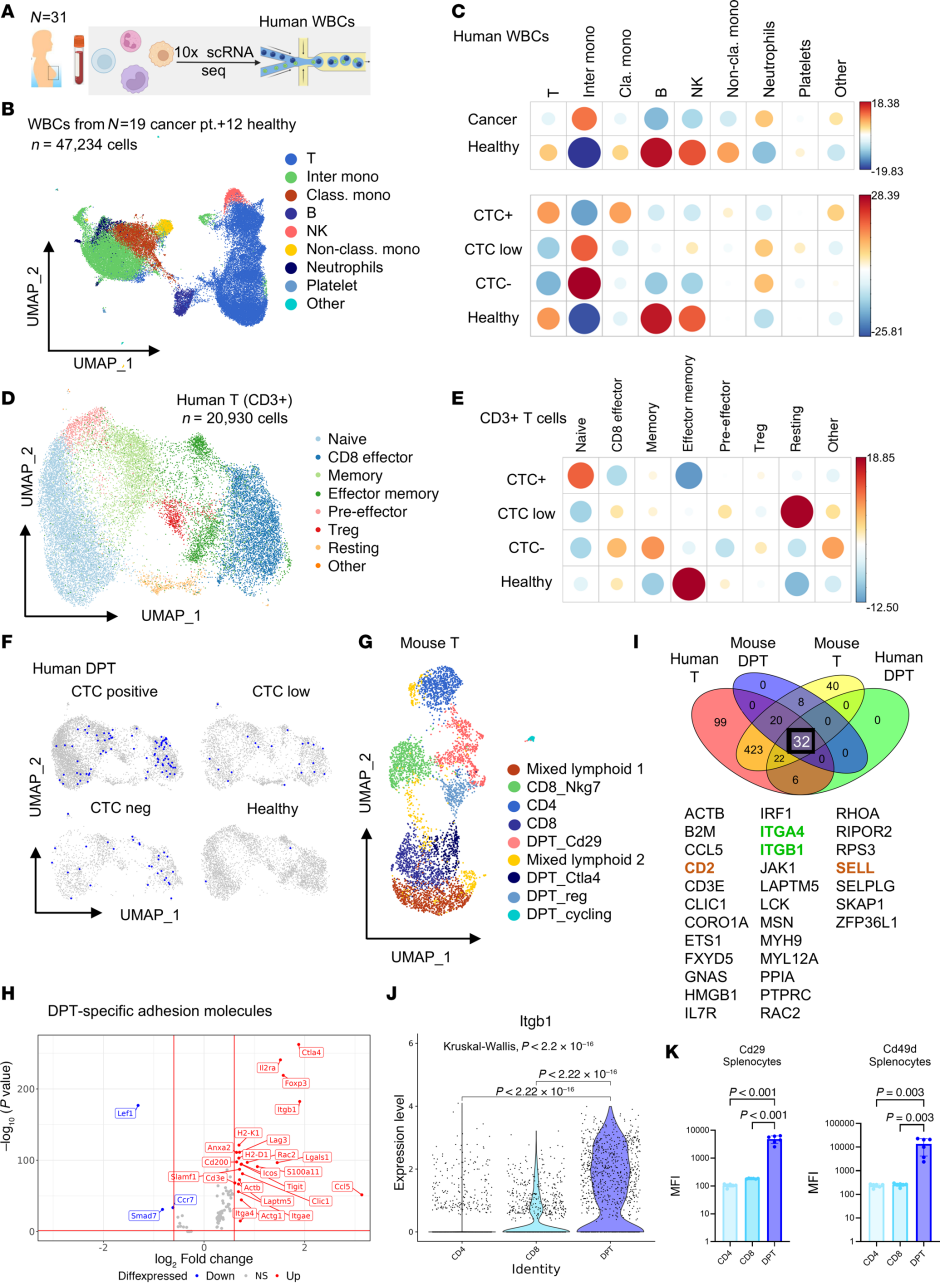
scRNA-Seq reveals enrichment of rare T cell subsets (DPTs) in the blood of breast cancer patients, dependent on CTC status. (**A**) Schematic depicting the isolation of human WBCs and subsequent scRNA-Seq. (**B**) UMAP plots of 47,234 single WBCs from 19 breast cancer patients (*n* = 35,401 cells) and 12 healthy control liquid biopsies (*n* = 11,833 cells), with broad immune cell subsets annotated. (**C**) Correlation plots depicting χ^2^ test residuals to determine over- or underenrichment of each immune cell subset from the WBCs of breast cancer patients versus healthy controls (top) and by CTC status (bottom). Dot size corresponds to the absolute value of correlation coefficients, and color corresponds to χ^2^ residuals. (**D**) UMAP plots of T cell subsets (*n* = 20,930 cells). (**E**) Correlation plots of over- or underenrichment of each T cell subset from the WBCs of CTC-positive, -low, and -negative cancer patients and healthy controls. (**F**) DPT cells highlighted on T cell subset UMAP plots, split by CTC status. (**G**) UMAP plot of mouse T cells, including single-positive and DPT cells collected from BALB/c mouse splenocytes. (**H**) Volcano plot depicting most differentially expressed genes in DPT cells versus all other T cells in mouse splenic T cells. (**I**) Venn diagram showing the overlap between adhesion molecule genes expressed in total human T, mouse T, human DPT, and mouse DPT cells and a list of 32 genes shared among 4 groups. (**J**) Violin plots of mouse *Itgb1* mRNA expression in mouse T cells isolated from splenocytes, as measured by scRNA-Seq. Kruskal-Wallis test *P* values are provided. (**K**) Bar graphs of Cd29 (encoded by *Itgb1*) and Cd49d (encoded by *Itga4*) expression (MFI) in mouse DPT, CD4^+^, and CD8^+^ cells isolated from BALB/c mouse splenocytes. One-way ANOVA with Tukey’s multiple-comparison test *P* values provided. *N* = 6 mice.

A previous study reported the presence of intratumoral DPT cells (39), yet circulating DPT cells are rarely studied compared with other WBCs. To bridge this gap, we performed scRNA-Seq of human peripheral WBCs from 19 breast cancer patients and 12 healthy controls (Figure 4A and Supplemental Table 5). To compare different patients, samples were multiplexed using hashtag oligonucleotides (HTOs). Each patient was labeled with a unique HTO per sequencing run for downstream identification. Additionally, since our samples were collected over the course of several months, the dataset was integrated to control batch effects among sequencing runs that contained multiple hash-tagged patient samples per run/batch and to facilitate comparative analysis across datasets (40). After performing quality control to remove low-quality cells and potential doublets followed by dataset integration, 8 broad immune cell types were identified in both cohorts (Figure 4B and Supplemental Figure 8, A and B). When comparing overall immune cell representation and expected abundance based on cancer status, several immune cell types demonstrated differences, including overrepresented T, B, and NK cells in healthy controls along with more abundant intermediate monocytes in breast cancer patients (Figure 4C and Supplemental Figure 9A, left). However, when the patients were stratified by their CTC status into 3 groups, WBCs from CTC-positive patients (≥5 CTCs) showed a higher proportion of T cells and classical monocytes than the other 2 cancer groups with low (1–5 CTCs) or no CTCs (Figure 4C, Supplemental Figure 8C, and Supplemental Figure 9A, right).

To further understand the T cell subset in general, and DPT cells specifically, we isolated the T cell cluster, reperformed dimensionality reduction, and annotated the resulting T cell subsets. Here, we identified 8 subsets of T cells based on their transcript expression, such as naive T cells (*TCF7*), CD8^+^ effector (*NKG7*), memory and effector memory (*IL7R*), and Tregs (*FOXP3*) (Figure 4D and Supplemental Figure 8D). While healthy controls had an enrichment in the abundance of effector memory T cells, CTC-positive cancer patients had overrepresented naive T cells (Figure 4E, Supplemental Figure 8, E and F, and Supplemental Figure 9B, left). In contrast, CTC-low and -negative patients possessed a relatively high abundance of circulating effector and memory T cells (Figure 4E and Supplemental Figure 9B, right), which might be able to reject CTCs upon encountering them. Consistent with the literature (41), Tregs were slightly overrepresented in cancer patients in our dataset analyses (Figure 4E).

Based on transcript expression cutoff values of *CD4* and *CD8A*, we identified DPT cells (~500 cells) that were distributed in the CD8^+^ effector, memory (mostly CD4^+^), and naive T subsets with a clear enrichment in CTC-positive patients (Figure 4F and Supplemental Figure 8G). Using a public scRNA-Seq database of peripheral blood cells from patients with TNBC (42), we extracted additional DPT cells for combined profiling with our data (a total of 1,454 DPT cells), alongside 1,000 single-positive CD4^+^ or CD8^+^ T cells from each dataset. In this combined dataset, we observed the heterogeneity of DPT cells, as some are present in scRNA-Seq clusters with CD8^+^ T cells, while others more closely cluster with CD4^+^ T cells (Supplemental Figure 8H, Supplemental Figure 10, A and B, and Supplemental Figure 11A). Furthermore, we conducted scRNA-Seq on sorted mouse splenic single-positive T and DPT cells. DPT cells are abundant in multiple clusters with relatively distinct profiles from single-positive T cells on the UMAP plot (Figure 4G). In the mouse dataset, DPT and single-positive T cells from tumor-naive or tumor-bearing mouse spleens were sorted and stained with HTOs separately. DPT or single-positive T cell identity for each cluster was determined based on both gene expression and abundance of each hashtag label present in those clusters (Supplemental Figure 10, C–E). In general, we observed negligible differences between DPT and T cells from tumor-bearing and tumor-naive mice. While DPT cells dominated some clusters (Supplemental Figure 10E), there was some shared gene expression between single-positive T and DPT cells, likely owing to the heterogeneity and plasticity of DPT cells originating from single-positive T cells. Compared with single-positive T cells, the mouse DPT cells show the strongest enrichment for immunosuppressive genes *Foxp3* and *Ctla4*, activation gene *Il2ra* (*Cd25*), and integrin *Itgb1*, along with moderately enriched *Itga4* (Figure 4H and Supplemental Figure 11B).

In search of the molecular candidates contributing to DPT-CTC clustering, we analyzed expression of genes within the KEGG cell adhesion molecules pathway genes (*n* = 598) (43) and identified 32 genes with overlapping expression among mouse and human T and DPT cells, including integrins *ITGB1* and *ITGA4*, *SELL*, and *CD2*, among others (Figure 4I). Notably, mouse *Itgb1* (protein product Cd29) and *Itga4* (protein product Cd49d), which together form the integrin heterodimer, very late antigen 4 (VLA-4), were among the most enriched molecules in DPT cells compared with single-positive T cells (Figure 4, H–J, Supplemental Figure 11C, and Supplemental Figure 12A). The higher expression levels of integrin proteins Cd29 and Cd49d were also observed in DPT cells relative to single-positive T cells from mouse spleen and blood, as demonstrated via flow cytometry analyses (Figure 4K and Supplemental Figure 12, B–D). Consistently, a similar but moderate enrichment of human *ITGB1* expression was also observed in blood DPT cells versus CD4^+^ and CD8^+^ T cells, based on scRNA-Seq data of breast cancer patients (Supplemental Figure 7E).

To determine the functional importance of these candidate genes in heterotypic T cell interaction with tumor cells, we conducted genetic modulations by transient transfection of Jurkat T cells (or breast cancer cells) that stably express Cas9 with 2 individual synthetic guide RNAs (sgRNAs) for each gene. We first knocked out *ITGB1* and *SELL* in Jurkat cells and confirmed their depletion via flow cytometry (Supplemental Figure 12F, left panels). As sgRNAs were unavailable for *CD2*, we alternatively knocked out *CD58*, the cognate ligand for CD2, in MDA-MB-231 breast cancer cells (Supplemental Figure 12F, right panel).

We then performed a surrogate screening test for heterotypic T tumor cell clustering in vitro, with 40,000 Jurkat cells (red) and 10,000 MDA-MB-231 cells (green) added to cell suspensions in a poly-HEMA–coated 96-well plate (Supplemental Figure 12, H and J). As monitored by Incucyte time-lapse imaging of clustering, among all tested gene modulations, *ITGB1*-KO in Jurkat cells via 2 separate sgRNAs showed the strongest inhibition of heterotypic T tumor cell interactions with differences present as early as 3 hours (Supplemental Figure 12, G and H). One sgRNA for *SELL*-KO in Jurkat cells also moderately reduced heterotypic interactions, whereas another *SELL* sgRNA-mediated KO in Jurkat cells or *CD58-*KO in MDA-MB-231 cells did not significantly impact heterotypic clustering (Supplemental Figure 12, G–J). Therefore, ITGB1 (VLA-4 in partnership with ITGA4) became the top candidate to be further investigated in mouse models in vivo.

### VCAM1 is required for CTC-DPT clustering in a spontaneous metastasis model.

**Figure 5:**
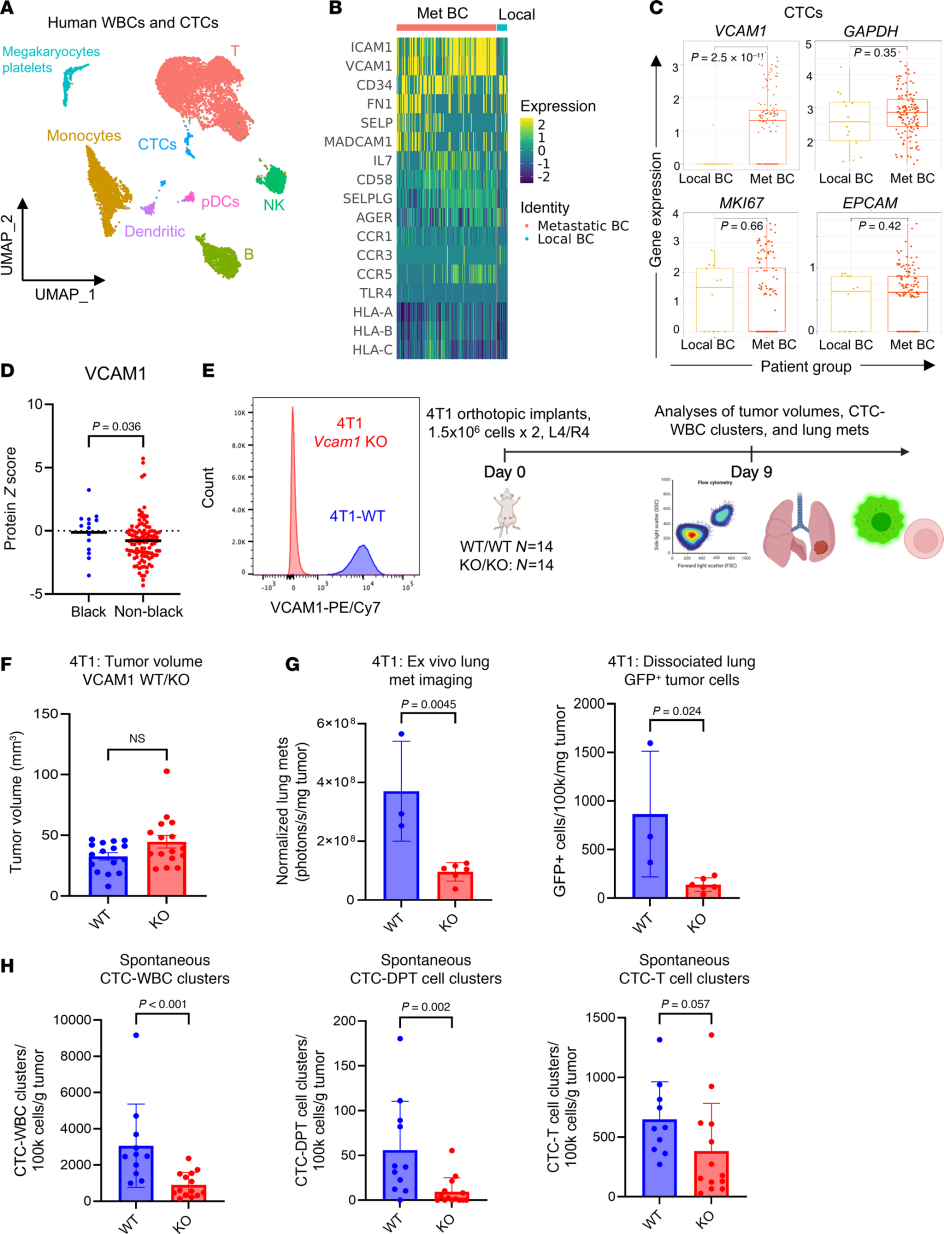
Targeting VCAM1 inhibits spontaneous lung metastasis and CTC-DPT clusters in vivo. (**A**) UMAP plot of CTCs and WBCs analyzed from a scRNA-Seq dataset of patients with breast cancer. (**B** and **C**) Heatmap of ligand adhesion molecules (**B**) and gene expression bar graph of *VCAM1* and control genes *GAPDH*, *MKI67*, and *EPCAM* via scRNA-Seq (**C**) in CTCs isolated from the patients with local and metastatic (Met) breast cancer (BC). Two-tailed unpaired *t* test. *N* = 138 for Met BC group and 14 for local BC group. (**D**) Plot of proteomic VCAM1 in nontreated breast cancer tumors of Black and non-Black patients. Unpaired, 1-tailed nonparametric *t* test. *N* = 14 for Black patients and 108 for non-Black. Black line on graph indicates median value. (**E**) Left: Flow histograms of mouse 4T1 tumor cells, WT, and *Vcam1*-KO. Right: Schematic depicting experimental design of orthotopic implants of eGFP^+^ 4T1 (WT and *Vcam1*-KO) tumors in BALB/c mice and subsequent analyses of tumor burden, CTCs, and lung metastasis. (**F**) Bar graphs of 4T1 primary tumor volumes, WT and *Vcam1*-KO, on day 9 (NS = not significantly changed, *P* > 0.05) prior to eGFP immunogen–triggered immune attacks in mice. *N* = 16 tumors. Two-tailed unpaired *t* test. (**G**) Bar graphs of lung metastatic signals of eGFP^+^ 4T1 cells detected via ex vivo fluorescence imaging of dissected lungs (left) and flow cytometry of dissociated eGFP^+^ cells from the mouse lungs (right). *N* = 3 for WT and 6 for KO. Unpaired, 2-tailed *t* test *P* values are displayed. (**H**) Bar graphs showing CTC-WBC clusters, CTC-T cell clusters, and CTC-DPT cell clusters in peripheral blood of mice with 4T1-NT control or VCAM1-KO tumors. *N* = 11 for WT and 14 for KO. Unpaired, 2-tailed *t* test *P* values are displayed.

To identify the ligand(s) in tumor cells that interacts with candidate receptors in DPT cells, we expanded the scRNA-Seq data with a publicly available dataset of peripheral WBCs from breast cancer patients (44) that include a subset of CTCs among the WBC populations (Figure 5A). This dataset includes 138 CTCs detected from 13 metastatic breast cancer patients and 14 CTCs detected from 1 local breast cancer patient (GSE139495). We analyzed the expression of predicted ligands on CTCs that would bind to adhesion molecules identified to be highly expressed in DPT cells (Figure 4I). From the heat map of predicted ligands in CTCs that could interact with the top adhesion molecules expressed in DPTs, we found 2 top candidates, *ICAM1* and *VCAM1*, with higher expression in CTCs from the patients with metastatic breast cancer than those from patients with local disease (Figure 5, B and C). Our previous work demonstrated that ICAM1 promotes cancer stemness and homotypic CTC cluster formation (14). We analyzed the mass spectrometry proteomic datasets of treatment-naive human breast primary tumors (45) (*n* = 122) and found a higher expression of the VCAM1 protein in Black patients with breast cancer versus non-Black patients (Figure 5D). As VCAM1 is a known ligand of VLA-4 and is involved in breast cancer metastasis (46, 47), we hypothesized that VCAM1 contributes to tumor cell–T cell interactions.

To test the hypothesis, we employed the 4T1 mouse syngeneic model and patient CTC-derived CTC-205 model, both of which develop spontaneous metastasis to the lungs. We knocked out *Vcam1* in stably expressing Cas9 4T1 cells, implanted them into the mammary fat pads of BALB/c mice, and allowed tumors to grow with CTCs detected as early as 9–16 days after implantation before immune recognition of luciferase and/or fluorescent protein labels lead to potential tumor rejection (Figure 5E). We harvested blood, lungs, and tumors and assessed CTC-WBC clusters, immune infiltrates in the tumor and lungs, and metastatic burden in these animals.

While primary tumor burden was not significantly altered between the 4T1-WT and *Vcam1-*KO groups (Figure 5F), significant decreases in metastatic signals were observed in the lungs of 4T1-*Vcam1*-KO mice, as detected by biofluorescent imaging and flow cytometry (Figure 5G and Supplemental Figure 14A). Additionally, *Vcam1-*KO in 4T1 cells reduced the formation of CTC-WBC clusters, nearly eliminating CTC-DPT cell clusters without altering other CTC-T cell clusters (Figure 5H). Interestingly, CTC clusters with granulocytic myeloid-derived suppressor cells (gMDSCs) also decreased in the blood of *Vcam1-*KO–bearing mice, while other heterotypic clusters with B cells, monocytic MDSCs (mMDSCs), and neutrophils did not change significantly (Supplemental Figure 13 and Supplemental Figure 14B).

In primary tumors, *Vcam1* KO caused a significant decrease in infiltrated CD3^+^ T cells, coupled with increased gMDSCs (Supplemental Figure 14C). A similar increase of gMDSCs was observed in the lungs of KO tumor-bearing mice (Supplemental Figure 14D), along with a decrease in circulating T cells, B cells, and mMDSCs in the peripheral blood of these mice (Supplemental Figure 14E). Together, these data show that Vcam1 on tumor cells is required for optimal CTC-DPT cell cluster formation and spontaneous lung metastasis in mice.

To determine if the metastatic phenotype was mediated by DPT cells independently of other immune cells, we repeated this experiment in NOD.Cg-*Prkdc^scid^* *Il2rg^tm1Wjl^*/SzJ (NSG) mice that lack T cells, among other immune cell subsets. In the immune compromised mice, *Vcam1*-KO primary tumors were markedly larger than WT counterparts (Supplemental Figure 15A). However, unlike in BALB/c mice that saw a reduction in lung metastasis, the lung metastatic signal, normalized to primary tumor volume, was increased in NSG mice bearing *Vcam1*-KO tumors (Supplemental Figure 15B). We did not observe any changes in single CTCs or CTC-WBC clusters (Supplemental Figure 15, C–E), suggesting that the reduced metastatic phenotype of *Vcam1*-KO tumor cells observed in immune competent mice is dependent on the presence of DPT cells.

Next, we wondered whether the CTC–DPT interaction was dependent on T cell receptor (TCR) recognition of major histocompatibility complex (MHC) molecules on CTCs, as genes involved in TCR/MHC antigen recognition (*B2M* and *HLA*) were highly expressed in human and mouse DPT cells (Figure 4, H and I). To address this question, we preincubated 4T1 cells with anti–MHC-I neutralizing antibodies and performed a clustering assay with mouse splenic CD3^+^ T cells. Anti–MHC-I treatment did not interfere with the heterotypic cluster formation in vitro (Supplemental Figure 16A). We repeated this experiment using splenic CD3^+^ T cells from mice with or without 4T1 tumors and again saw no difference in heterotypic cluster formation between the groups (Supplemental Figure 16B).

To verify the role of VCAM1–VLA-4 interactions in human breast cancer, we profiled VCAM1 expression in cell lines and multiple patient-derived xenograft (PDXs) models (such as TN1 and CTC-derived model CTC-205) we generated (32, 37). Our previous work identified VCAM1 was 1 of the top 2 enriched genes (along with ICAM1 and the stemness signature) in the lung metastases of PDXs compared with primary tumor cells (14). We further confirmed the higher expression of VCAM1 in spontaneous metastases of PDXs (CTC-205), with minimal VCAM1 expression in primary tumors (Supplemental Figure 16C).

Compared with the VCAM1^–^ tumor cells from the primary tumor xenograft (CTC-205), VCAM1^+^ tumor cells sorted from the lung metastases (CTC-205-Met-VCAM1) promoted heterotypic clustering with WT Jurkat cells. However, this interaction was disrupted when the tumor cells were mixed with *ITGB1*-KO Jurkat cells (Supplemental Figure 16, D and E). These findings suggest that the human tumor cell–T cell interactions also depend on a molecular network involving VCAM1 on tumor cells and ITGB1 on T cells.

### Targeting the VLA-4 axis reduces CTC-DPT clusters and lung metastatic burden.

To assess whether targeting VLA-4 could be translated into a therapy for treating metastatic breast cancer, we investigated the use of neutralizing antibodies. We hypothesized that anti–VLA-4 treatment would reduce CTC-DPT clusters and thus limit metastasis.

**Figure 6:**
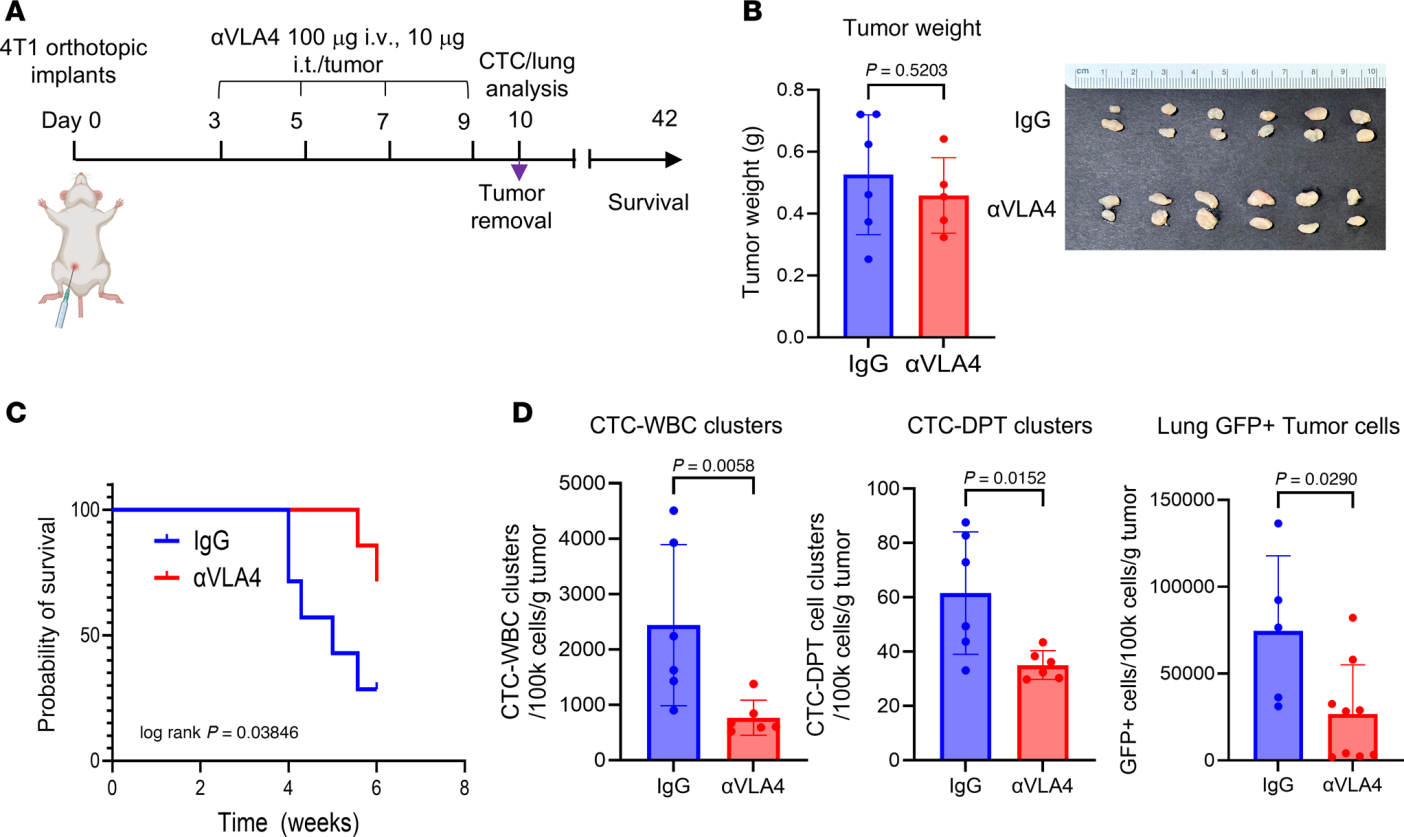
Targeting VLA-4 inhibits spontaneous lung metastasis and CTC-DPT clusters in vivo. (**A**) Schematic of anti–VLA-4 neutralizing antibody (αVLA-4 Ab) treatment for orthotopic 4T1 breast tumors on days 3, 5, 7, and 9. Blood/tissue harvests for primary tumors, CTCs, and lung metastasis analyses on day 10 (*N* = 6). The primary tumor was removed via survival surgeries with the rest of the mice (*N* = 7 per group) for extended survival analyses until 6 weeks. (**B**) Bar graphs of 4T1 tumor weight and photos of dissected tumors of IgG control and αVLA-4–treated groups on day 10. Unpaired 2-tailed t test (*N* = 6) between 2 groups. (**C**) Mouse survival after neoadjuvant treatment of IgG and αVLA-4 followed by a surgical removal of primary tumors on day 10 (*N* = 7). Log-rank *P* = 0.03846 for distinct survival of 2 groups of the mice by 6 weeks. Hazard ratio = 4.435 (0.9842–20.75, 95% CI of ratio) when the IgG group was compared with the αVLA-4–treated group. (**D**) Bar graphs of blood-detected CTC-WBC clusters (left) and CTC-DPT clusters (middle), and the burden of lung tumor cells (right) detected and quantified via flow cytometry on day 10 (*N* = 6). Unpaired, 2-tailed *t* test *P* values are shown.

To test this, we implanted orthotopic 4T1 tumors into mice for neoadjuvant treatment with anti–α4-integrin (100 μg) via tail vein injection, plus 10 μg per tumor in the tumor bed, beginning on day 3 and continuing every other day for a total of 4 treatments before surgical resection of primary tumors on day 10 (Figure 6A). This regimen did not significantly affect the primary tumor size (Figure 6B). Remarkably, anti–VLA-4 treatment significantly extended the animal survival (Figure 6C). The blood and lung analyses on day 10 revealed decreased CTC-WBC clusters and CTC-DPT cell clusters, as well as the number of tumor cells in the lungs in the mice treated with anti–VLA-4 (Figure 6D). Notably, we observed no significant differences in the immune cell populations within circulation, the primary tumor, or the lungs between the control and treated groups (Supplemental Figure 17, A–D).

Collectively, these data suggest that blocking VLA-4–mediated interactions and clustering of WBC/DPT and CTC offers what we believe to be a novel strategy to reduce the metastatic burden in breast cancer.

## Discussion

By integrating CTC analyses from a large cohort of breast cancer patients with mechanistic studies, our work reports what we believe to be a novel discovery of heterotypic CTC-WBC clusters containing a rare subset of DPT cells with unique features of exhaustion and immune suppression (TIM-3 and FoxP3), thereby fostering tumor pluripotency and immune evasion. DPT cells have been observed in association with many diseases, such as increased frequencies in the periphery and tumor infiltrates of various cancers (39, 48–53). We demonstrate that DPT-CTC clusters drive breast cancer metastasis via VLA-4 (ITGA4/B1)–VCAM1 interactions. Notably, upregulated VCAM1 expression was observed in primary breast cancer of Black patients along with a higher frequency of heterotypic CTC-WBC clusters than White patients. This finding is positively associated with clinical outcome disparity of breast cancer, especially TNBC, with a disproportionately higher mortality risk for Black patients than for White and Asian patients (54–57), mainly due to metastasis.

To effectively prevent and eliminate metastasis, it is necessary to target primary tumors, CTCs (single CTCs, homotypic clusters and diverse heterotypic clusters), and metastases in distant organs. In addition to targeting homotypic CTC clusters (6, 14–21, 58), one of the pivotal therapeutic directions would be interfering with CTC-WBC clusters. As proof of concept, targeting VLA-4 (α4) disrupts the VCAM1–VLA-4 interactions between CTC and immunosuppressive DPT and inhibits metastasis, ultimately improving breast cancer outcomes. Beyond cancer diseases, targeting VLA-4 has been safely and effectively extended to treat Crohn disease and multiple sclerosis (59). Meanwhile, it is speculated that immune checkpoint inhibitors such as anti–PD-1 and anti-TIM3 would beneficially boost antitumor immunity for advantageous clearance of CTCs in close contact.

Our data suggest that the inhibited metastatic phenotype in VCAM1-KO tumor cells involves DPT cells, whereas in immunocompromised NSG mice, CTC-WBC clusters or spontaneous metastasis to the lungs do not decrease. Please note that NSG mice are not specifically deficient in DPT cells, but also lack single-positive T cells, B cells, and functional NK cells, which may also influence the observed metastatic phenotype. It is necessary to investigate this effect in a mouse model lacking only DPT cells. However, that is currently not feasible until unique markers of DPT cells are identified for depletion.

In addition to DPT cells, we have detected other immune cells, such as neutrophils, monocytes, NK cells, and B cells, in CTC-WBC clusters. Overall CTC-WBC clusters decrease in mice bearing 4T1-*Vcam1*-KO tumors and after anti–VLA-4 treatment. Beyond DPT-mediated effects, some WBC subsets may rely on VCAM1–VLA-4 interactions directly (47, 60). Additionally, VCAM1 may bind to ligands such as integrin α4β7 and galectin-3 (61, 62) in lymphocytes, NK cells, myeloid cells, and macrophages (63–65). These interactions could be investigated for therapeutic targeting. When they are incapable of removing tumor cells, neutrophils (66) and monocytes are known to promote metastasis (32, 60, 66–68). NK cells (69), platelets (21, 60, 70), and MDSCs (32, 71) could enhance tumor cell seeding and metastasis. Furthermore, strategies for specific targeting of CTCs and homotypic CTC-CTC clusters via antibodies or antibody-drug conjugates must be exploited to inactivate drivers of cancer stemness and CTC clustering, such as ICAM1 (14), thereby blocking seeding and eliminating metastasis. Therefore, joint targeting efforts with cutting-edge technologies will accelerate the future reciprocal translation between the bench and the bedside.

Despite the robustness of our study, we recognize some technical limitations that may represent confounding variables and influence our results. It is well known that GFP and/or luciferase-expressing tumor cells can elicit an immune response against these exogenous proteins (72). We have attempted to control this variable by harvesting tissue before a full immune cell response led to primary tumor regression (usually on day 11). Furthermore, the observed GFP and luciferase signals in the primary tumors, CTCs, and lung metastases indicate that selective populations of GFP/luciferase-expressing cells have escaped from the immune response. However, a possible variable between non-GFP/luciferase and GFP/luciferase-expressing cells might influence the readout data from CTCs, CTC-WBC clusters, or lung metastases.

Furthermore, this study has focused primarily on lung metastasis, as both human and mouse TNBC models develop metastasis to the lungs only at tested time points. Consistently, the 4T1 model is known to metastasize to the lungs more frequently than other organ sites (73). We speculate that VCAM1 KO or anti–VLA-4 antibody treatment may alter CTC cluster–driven metastasis to other sites.

## Methods

### Sex as a biological variable

Our study mostly analyzed blood biospecimens from female patients, who account for over 99% of the patient population in breast cancer. Therefore, we exclusively examined female mice because the disease modeled is most relevant in females.

### Human sample analysis

CTCs (singles and clusters) of the blood samples collected in CellSave tubes (Menarini Silicon Biosystems) were analyzed via CellSearch (Menarini Silicon Biosystems), and those in EDTA tubes (BD Vacutainer) were analyzed via flow cytometry, CellView (BD Biosciences), and ImageStream (Cytek).

### Animal studies

All mice used in this study were housed in specific pathogen-free facilities, with a regular diet, regular light/dark cycles, and regular ambient temperature and humidity at the Center for Comparative Medicine at Northwestern University. For syngeneic models, female BALB/c mice aged 8–12 weeks (The Jackson Laboratory, strain 000651) or female NSG mice aged 8–12 weeks (The Jackson Laboratory, strain 005557) were randomized by age for orthotopic implantation with murine 4T1 cell line, human MDA-MB-231 cell line, or multiple TNBC PDX models.

### Antibody treatments

Anti–VLA-4 (BioXCell, catalog BE0071) or IgG control antibody (BioXCell, catalog BE0090) was diluted to a final concentration of 500 μg/mL in PBS. Animals were injected with up to 100 μg of antibody via the tail vein and up to 10 μg of antibody into the tumor bed via subcutaneous injection. Each mouse received treatment every other day 4 times. Further antibody information is provided in Supplemental Table 7.

### Breast tumor models and transfections

Murine 4T1, human MDA-MB-231, and Jurkat cells were acquired from ATCC and regularly tested for mycoplasma using the MycoAlert Mycoplasma Detection Kit (Lonza, catalog LT07-318). The PDX-CTC-205 model was generated from CTCs of a breast cancer patient as described previously (37). Early passages of cells (<20 passages) were cultured in RPMI (4T1 and Jurkat) or high-glucose DMEM (MDA-MB-231) supplemented with 10% FBS and 1% penicillin/streptomycin.

#### Cas9 transfection and CRISPR knockouts.

For lentivirus production, HEK293T cells at 70%–80% confluence were cotransfected with LentiCas9-EGFP (Addgene, plasmid 63592) and envelope vectors (pMD2.G and psPAX2) using PEI reagent (Polysciences, catalog 23966). The supernatant was harvested 48 and 72 hours after transfection, filtered with a 0.45 μm polyethersulfone (PES) filter, centrifuged at 100,000*g* for 2 hours, aliquoted, and stored at –80°C. Then, 3 × 10^5^ MDA-MB-231, 5 × 10^5^ Jurkat, or 2 × 10^5^ 4T1 cells were cultured in 6-well plates and transduced with concentrated lentiviral supernatant (MOI of 1) using polybrene (8 μg/mL). After 24 hours, the media containing lentivirus supernatant were replaced with fresh media. Cells were cultured 5 more days and then eGFP^+^ cells were separated using FACS.

To generate CRISPR/Cas9-mediated knockouts, TrueGuide sgRNAs (Thermo Fisher Scientific) were resuspended in 1× Tris-EDTA (TE) buffer at a concentration of 100 μM. Stock solution of sgRNA was stored at –20°C until further use. The day before transfection with sgRNA, Cas9-expressing cells were plated in a 6-well plate at a concentration of 250,000 cells/well. The next day, 37.5 pmol of sgRNA was mixed with 125 μL of Opti-MEM I reduced serum medium (Thermo Fisher Scientific, catalog 31985062) per transfection well. Lipofectamine CRISPRMAX Cas9 transfection reagent (7.5 μL; Thermo Fisher Scientific, catalog CMAX00001) was added to 125 μL Opti-MEM I medium per transfection well and incubated at room temperature for 5 minutes. The diluted transfection reagent solution was added to the tube containing the sgRNA solution and mixed by pipetting. This solution was incubated for 15 minutes at room temperature, and 250 μL of the sgRNA/transfection reagent complex was added to each of the wells. Cells were allowed to grow for 2–3 days, upon which they were harvested, stained for the protein targeted by the sgRNA, and analyzed by flow cytometry. Cells negative for the protein were sorted using a FACSAria sorter (BD Biosciences), replated, and propagated in vitro. Cells were then stained for flow cytometry, and single cells were sorted into 96-well plates to generate clones. Knockout efficiency of clones was determined via protein analysis, and individual and pooled clones were sorted for downstream use.

### CTC analyses by CellSearch and flow cytometry

#### CellSearch.

The CellSearch system (Menarini Silicon Biosystems) is semiautomated for blood sample processing, enrichment of EpCAM^+^ epithelial CTCs using the Epithelial Cell Kit, and subsequent 4-channel immunofluorescence staining, as described by Cristofanilli et al. (5) CTCs are specified by combining 3 routine channels of DAPI^+^, CK^+^, and CD45^–^. Single CTCs, homotypic CTC clusters, and heterotypic CTC clusters were manually verified and counted.

#### Flow cytometry.

Patient blood samples were collected in EDTA Vacutainer tubes (Becton Dickinson) and stored on ice for processing within 24 hours after collection. Samples were spun at 300*g* for 10 minutes with no brake to remove plasma, which was stored at –80°C. Remaining blood cells were subject to 2–4 rounds of red blood cell lysis using Red Blood Cell Lysing Buffer Hybri-Max (Sigma-Aldrich, catalog R7757). WBCs were washed in PBS and resuspended in PBS with 2% FBS. Cells were blocked using mouse IgG (Bio-Rad, catalog PMP01) for 10 minutes on ice and stained with antibodies for EpCAM, CD45, CD3, CD4, CD8, CD19, CD11b, CD14, CD16, CD56, and CD66b on ice, protected from light, for 30 minutes. Cells were washed with PBS and resuspended in PBS with 2% FBS, stained with DAPI or Live/Dead Fixable Blue Dye (Thermo Fisher Scientific, catalog L23105), and analyzed on an LSRFortessa analyzer (BD Biosciences). For ImageStream analysis, samples were run on a Cytek Amnis ImageStream^X^ Mk II imaging flow cytometer (Cytek Biosciences) and analyzed using IDEAS image analysis software version 6.2.

### Splenocyte and T/DPT cell collection

Female BALB/c mice were sacrificed by CO_2_ asphyxiation followed by cervical dislocation. Spleens were removed and forced through a 70 μm mesh filter (Thermo Fisher Scientific, catalog 22363548) using the plunger of a 3 mL syringe (Thermo Fisher Scientific, catalog 14823435). Cells were washed in PBS, spun at 300*g* for 5 minutes, and resuspended in red blood cell lysis buffer. Cells were incubated in lysis buffer on ice for 5 minutes, washed in PBS to stop the reaction, and spun at 300*g* for 5 minutes. Cells were resuspended in PBS with 2% FBS and 20 mM EDTA, filtered through a 40 μm mesh filter (Thermo Fisher Scientific, catalog 22363547), and counted. The MojoSort Mouse CD3 T Cell Isolation Kit (BioLegend, catalog 480024) was used to isolate T/DPT cells. Briefly, unlysed splenocytes were incubated with the biotin-antibody cocktail (BioLegend) for 10 minutes on ice, followed by the Streptavidin Nanobeads (BioLegend) for 10 minutes on ice. Cells of interest were separated using the MojoSort Magnet (BioLegend, catalog 480019) for 2 separations. Cells were washed in PBS and resuspended in PBS with 2% FBS and 20 mM EDTA. To further isolate specific T cell populations (CD4, CD8, or DPT), cells were blocked with TruStain FcX (anti-mouse CD16/CD32) antibody (BioLegend, catalog 101319) for 10 minutes on ice and stained with antibodies against mouse CD45, CD3, CD4, and CD8. Samples were sorted on a FACSAria SORP system (BD Biosciences) or a Miltenyi MACSQUANT Tyto Cell Sorter using MACSQUANT Tyto Cartridges HS (Miltenyi Biotec, catalog 130121549) for downstream use.

### Clustering assay

For cell lines, 96-well plates were coated with poly-HEMA (Sigma-Aldrich, catalog P3932) overnight at room temperature and then washed with PBS. For PDX cells, 96-well plates were coated in collagen I, bovine (Corning, catalog 354321), diluted 1:100 in water for 1 hour at room temperature, and washed with PBS. Cells were stained with PKH67 Green Fluorescent Cell Linker (Sigma-Aldrich, catalog PKH67GL) or Incucyte Cytolight Red (Sartorius, catalog 4706) according to the manufacturers’ instructions. Single-cell suspensions of 4T1, MDA-MB-231, PDX, or immune cells were added to the wells at a final volume of 200 μL. Plates were imaged using the Incucyte ZOOM Live Cell Imaging System (Essen Biosciences) and analyzed using the Incucyte ZOOM Software. For the metastasis lung colonization assay, after clustering, cells were gently collected into an EasyTouch U-100 insulin syringe (MHC Medical Products, catalog 829155), so as not to disturb clusters, and injected into the tail veins of BALB/c mice.

### Bioluminescent imaging

Animals were injected i.p. with 100 μL d-luciferin at 30 mg/mL (Gold Biotechnology, catalog 115144359), anesthetized using isoflurane, and placed into the In Vivo Imaging System Lago X (Spectral Instruments Imaging). Bioluminescence was detected with an acquisition time up to 5 minutes. The 0-hour time points indicate images collected immediately (up to 10 minutes) after cells were injected. Images were analyzed using Aura In Vivo Imaging Software (Spectral Instruments Imaging). Signal is reported as total flux (photons/second) and, when necessary, normalized to the 0-hour (10 minutes) time point on a per-animal basis.

### IHC

Tissues were fixed in 4% paraformaldehyde for 24–48 hours and transferred to 70% ethanol solution. Tissue was embedded in paraffin blocks, cut into 4-μm-thick sections, and processed for H&E or IHC staining by the Mouse Histology and Phenotyping Laboratory at Northwestern University.

### Cell isolation and scRNA-Seq library preparation

Bulk WBCs or mouse splenic T cells were processed as described above and CD45^+^ single cells or mouse T/DPT cells were sorted into tubes of PBS with 2% FBS on a BD FACSAria sorter. To collect mouse splenic DPT and single-positive T cells, spleens from 4T1 tumor–bearing or tumor-naive BALB/c were processed and sorted separately before staining with multiplexing hashtags. DPT cells from each mouse group were sorted into 1 tube, and single-positive T cells were sorted into another, and each were stained for multiplexing separately. For 4T1 clustering samples, 4T1-DPT or 4T1-splenocyte clusters were generated as described above, collected, and washed with PBS. The clusters were gently disturbed to generate single-cell suspensions before proceeding to the next step. For experiments with multiplexing, sorted cells were blocked with Human TruStain FcX (Fc Receptor Blocking Solution; BioLegend; catalog 422301) or TruStain FcX (anti-mouse CD16/32) antibody (BioLegend; catalog 101319) for 10 minutes on ice, followed by staining with TotalSeq-C anti-human or anti-mouse hashtag antibodies for 30 minutes on ice. For multiplexing, after sorting, each patient was labeled with a unique hashtag antibody for downstream identification. In mouse experiments, cells from the same conditions were labeled with unique hashtags (e.g., DPT naive, DPT tumor, T naive, and T tumor). Cells were washed 3 times in Cell Staining Buffer (BioLegend; catalog 420201) and resuspended in PBS with 2% FBS in a single-cell suspension. The concentration and viability of the single-cell suspensions were measured using a Cellometer Auto 2000 with ViaStain AOPI staining solution (Nexcelom, catalog CS2-0106). Cells were loaded onto a 10X Chromium Controller for GEM generation followed by single-cell library construction using the 10X Chromium Next GEM Single Cell 5′ Library and gel bead kit v1.1 (10X Genomics, catalog PN-1000165) according to the manufacturer’s protocol. Quality control of the libraries was performed using an Invitrogen Qubit DNA high sensitivity kit (Thermo Fisher Scientific, catalog Q32851) and an Agilent Bioanalyzer high-sensitivity DNA kit (catalog 5067-4626). The libraries were pooled in equal molar ratios and sequenced on an Illumina HiSeq 4000 using sequencing parameters indicated by the manufacturer (read 1: 26 cycles, i7 index: 8 cycles; read 2: 91 cycles).

### Generation of single-cell gene expression matrices

Demultiplexing, alignment, and gene quantification were all performed using Cell Ranger (10X Genomics, version 7.0.1). The run data were demultiplexed using cellranger mkfastq, and the resulting FASTQ files were aligned and counted using the 10X Genomics prebuilt human (refdata-gex-GRCh38-2020-A) or mouse (refdata-gex-mm10-2020-A) reference genomes.

### Quality control, cell-type clustering, and major cell type identification

For each sequencing run, 5% quantiles of genes expressed (features) and unique molecular identifiers (UMIs, counts) were calculated. Cells expressing less than 500 genes and UMIs were considered low quality and removed from the dataset. To remove doublets or clusters, cells that had features or counts greater than the 90th percentile for each given sequencing run were excluded from the analysis. Additionally, after demultiplexing, cells that were identified to be labeled with 2 different hashtag antibodies were considered doublets and excluded from the analysis. Demultiplexing was performed using the MULTIseqDemux function in the Seurat R package. Furthermore, cells with greater than 10% mitochondrial content were also excluded. Datasets were integrated (40) to control batch effects among multiple sequencing runs (multiple hash-tagged samples per run; detailed information is provided in Supplemental Table 6). Integration was performed using the FindIntegrationAnchors and IntegrateData functions in the Seurat R package to minimize batch variations (40). Dimensionality reduction and unsupervised cell clustering were performed using methods from the Seurat software suite (version 4.3.2). The results are presented using UMAP, and 19 distinct clusters were originally identified. After gene expression analysis, Seurat-determined clusters were collapsed into biologically relevant clusters that align with cell type. Cell types were identified by expression of CD3, CD14, CD16, CD19, NCR1, and FCGR2A, and other markers were identified by utilizing the FindMarkers () function in the Seurat package. T cell clusters were split from the main dataset and analyzed separately. To identify DPT cells, cells that had normalized RNA expression of both CD4 and CD8A greater than 0.2 from this dataset and from a public dataset (GSE169246) were selected. Additionally, 1,000 random CD4^+^CD8A^–^ and CD4^–^CD8A^+^ cells from each dataset were selected to generate a T/DPT only dataset for analysis. For differential gene expression analysis, cell identities were selected based on annotated gene expression (for instance, T vs. classical monocytes or DPT vs. CD4^+^) or based on hashtag expression (for example, DPT vs. splenocyte). Pathway expression was carried out using the Enrichr package (38).

### Statistics

GraphPad Prism 10 was used to perform statistical tests. For all tests, a *P* value of less than 0.05 was considered significant. R (version 4.4.0, https://www.r-project.org/) and Seurat (version 4.4.2, https://satijalab.org/seurat/), dplyr (version 1.1.4, https://dplyr.tidyverse.org/), tidyverse (version 2.0.0, https://www.tidyverse.org/), ggplot2 (version 3.5.1, https://ggplot2.tidyverse.org/), survminer (version 0.4.9, https://cran.r-project.org/web/packages/survminer/index.html), Enrichr (version 3.3, https://maayanlab.cloud/Enrichr/), dittoSeq (version 1.16.0, https://www.bioconductor.org/packages/release/bioc/html/dittoSeq.html), EnhancedVolcano (version 1.27.0, https://github.com/kevinblighe/EnhancedVolcano), and survival (version 3.7-0, https://cran.r-project.org/web/packages/survival/index.html) packages were used for data analysis and to generate figures. FlowJo software (version 10.10.0, https://www.flowjo.com/) was used to generate flow cytometry plots and for data analysis. All data in graphs represent mean ± SD unless otherwise specified.

### Study approval

Human blood sample collection and analyses were approved by the Northwestern University IRB (protocols STU00203283 and STU00214936) following NIH guidelines for human subject studies. Written consent was obtained from all participants whose blood samples were analyzed for the study. All animal procedures conformed to the NIH *Guidelines for the Care and Use of Laboratory Animals* (National Academies Press, 2011) (IACUC protocols IS00014098 and IS00021741) and were accepted by the Northwestern University IACUC.

### Data availability

The data supporting the findings of this study are available in the Supporting Data Values file. Human scRNA-Seq datasets generated in this study are available in NCBI’s Gene Expression Omnibus (GEO GSE249399): https://www.ncbi.nlm.nih.gov/geo/query/acc.cgi?acc=GSE294399 Mouse scRNA-Seq datasets generated in this study are available from the corresponding author upon reasonable request. Datasets GSE169246 and GSE139495 are available at https://www.ncbi.nlm.nih.gov/geo/query/acc.cgi?acc=GSE169246 and https://www.ncbi.nlm.nih.gov/geo/query/acc.cgi?acc=GSE139495 Human spleen immunofluorescence images are based on data generated by the HuBMAP Program (35) (https://hubmapconsortium.org).

#### Inclusion and diversity.

We are committed to fostering an inclusive research environment. Our study design and data analyses were structured to minimize biases and ensure equitable representation across relevant participant groups. Where possible, we stratified results to address biological and clinical variables such as sex, race (Asian, Black, White, and unknown race), and ethnicity, respecting privacy and ethical constraints. Researchers from diverse backgrounds and expertise were involved throughout the project, reflecting our commitment to multidisciplinary collaboration and inclusive scientific practices.

## Author contributions

DS and HL conceived the study and wrote the manuscript. DS completed the experiments and data analyses with support from LES, YJ, YZ, EH, CR, ADH, HFA, FT, JRS, and NKD. The senior authors HL, DF, MC, CHW, WJG, LCP, and JL supervised experimental planning and implementation. LES assisted with flow cytometry analyses of immune cells (WBCs) and CTCs (singles and clusters). LES, YJ, HFA, and NKD helped with animal work in vivo. YZ and CR collected CTC data from patients. EH assisted with blood sample processing and data analyses. ADH, FT, and YS facilitated the analysis of single-cell and proteomic data.

## Supplementary Material

Supplemental data

Supplemental table 3

Supplemental table 4

Supplemental table 5

Supplemental table 6

Supplemental table 7

Supporting data values

## Version 1 09/16/2025

Electronic publication
