# Supplementary material for: Double-positive T cells form heterotypic clusters with circulating tumor cells to foster cancer metastasis
Source: J Clin Invest. 2025 Sep 16;135(18):e193521. doi: 10.1172/JCI193521 (PMC12435850; doi:10.1172/JCI193521)
Supplement: Supplemental data [file jci-135-193521-s265.pdf]

Extended Table 1-2 and Figures S1-17

Supplemental Table 1. Demographic features of the patients with breast cancer

**Race**				
Asian	Black or African American	White	Unknown	
3.88% (17)	15.07% (66)	79.09% (307)	10.96% (48)	
**Age**				
< 55 years	> 55 years			
50.53% (190)	49.47% (186)			
**Stage**				
III	IV			
10.93% (46)	89.07% (375)			
**Molecular Subtype**				
Luminal A	Luminal B	HER2-enriched	Triple Negative	Unknown
37.67% (165)	15.98% (70)	20.32% (89)	25.57% (112)	0.46% (2)
**Received Chemotherapy?**				
Yes	No			
93.25% (373)	6.75% (27)			
**Received Endocrine Therapy?**				
Yes	No			
72.89% (285)	27.11% (106)			
**Received Anti-HER2 therapy?**				
Yes	No			
41.67% (160)	58.33% (224)			
**Received Immunotherapy?**				
Yes	No			
21.84% (88)	78.16% (315)			

Supplemental Table 2. Representative WBCs with surface markers used to identify immune cell types in CTC-WBC clusters

Cell Type	Markers
T cells	$CD45^{+}CD3^{+}CD4^{+/-}CD8^{+/-}$
B cells	$CD45^{+}CD3^{-}CD19^{+}$
NK cells	$CD45^{+}CD3^{-}CD16^{+}CD56^{+}$
Monocytes	$CD45^{+}CD3^{-}CD11b^{+}CD16^{+/-}CD14^{+/-}$
Neutrophils	$CD45^{+}CD3^{-}CD16^{+}CD66b^{+}$

Supplemental Figure 1

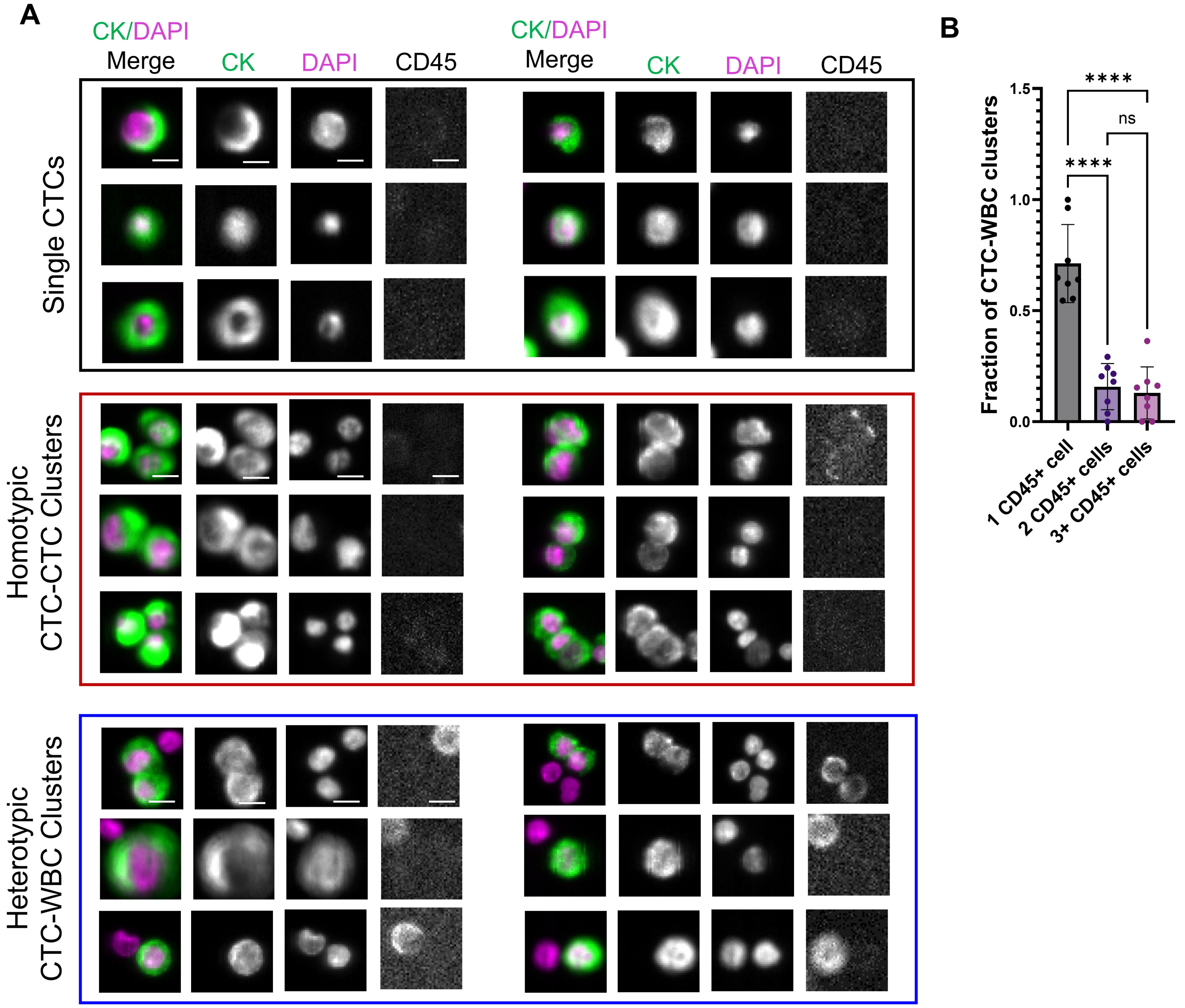

Supplemental Figure 1. Human CTCs (singles and clusters) from breast cancer patients detected via CellSearch.

A. Representative images of CTCs, singles, homotypic CTC-CTC clusters, and heterotypic CTC-WBC clusters with merged or single channels of cytokeratin (CK, green), DAPI (magenta), and CD45. Top panels: Single CTCs. Middle panels**.** Homotypic CTC clusters. Bottom panels: Heterotypic CTC-WBC clusters. Scale bar = 10 µm

B. Frequency of immune cell or WBC counts per CTC-WBC cluster. One-way ANOVA with Tukey's multiple comparison test; ****, $p < 0.001$. N = 8 CellSearch scans (patient biospecimens).

New Supplemental Figure 2

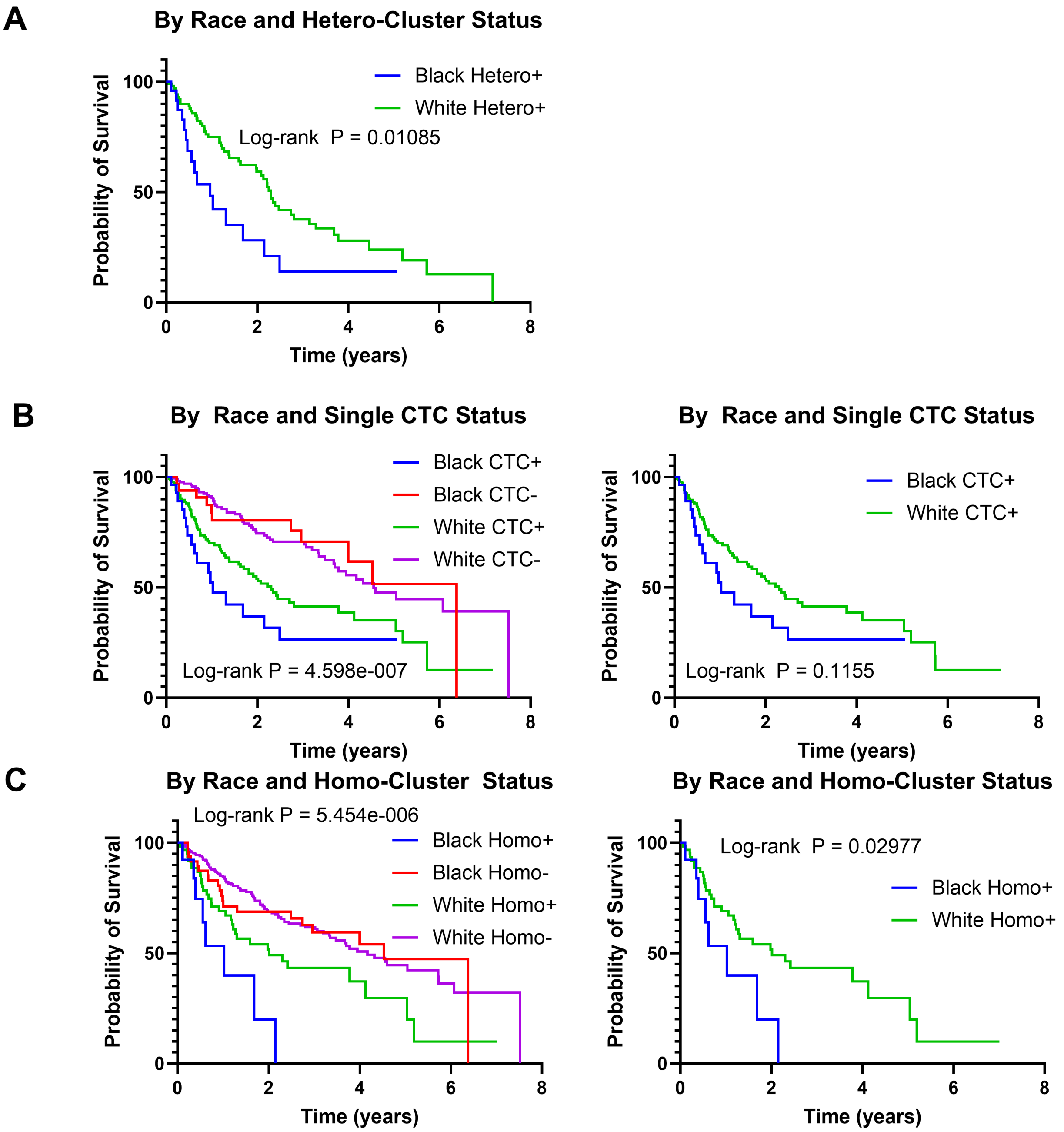

Supplemental Figure 2. Survival of Black and White patients stratified by CTC status.
A-C. Kaplan-Meier survival curves of patients with breast cancer, divided by race (Black and White) and heterotypic cluster status (**A**), single CTC status (**B**) homotypic cluster status (**C**). Log-rank (Mantel-Cox) test p-value shown.

Supplemental Figure 3

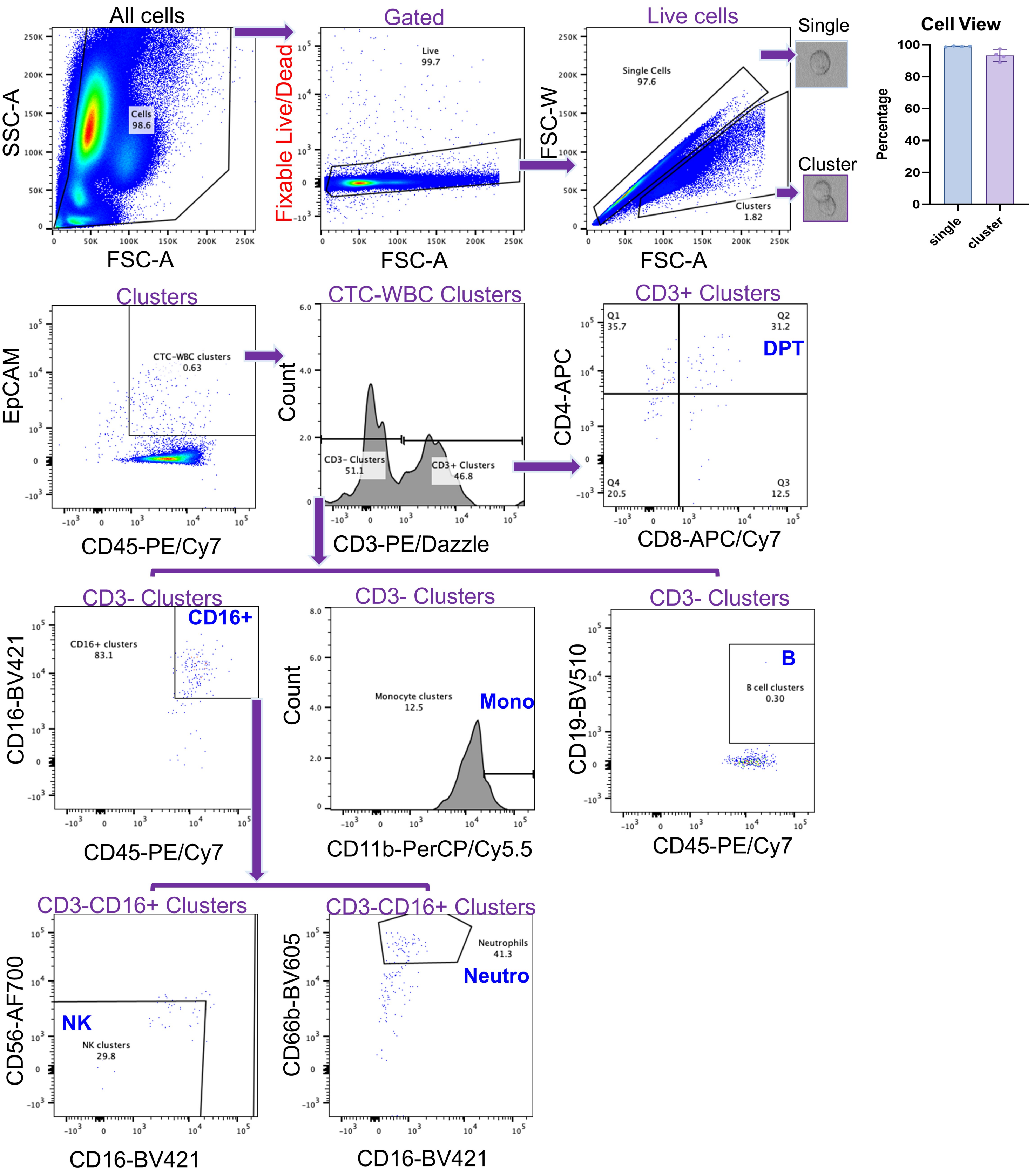

Supplemental Figure 3. Representative gating of CTC-WBC clusters. Single cells and clusters were gated based on forward/side scatter to distinguish their distinct sizes at an accuracy of 99% and 95%, respectively, as validated by BD CellView. WBC-CTC clusters were gated on CD45+EpCAM+, with $CD3^{+}$ T cell clusters, other $CD3^{-}$ clusters for B cells ($CD19^{+}$); NK cells ($CD16^{+}CD56^{+}$); neutrophils ($CD16^{+}CD66b^{+}$); and monocytes ($CD11b^{+}CD16^{+/-}$ $CD14^{+/-}$).

Supplemental Figure 4

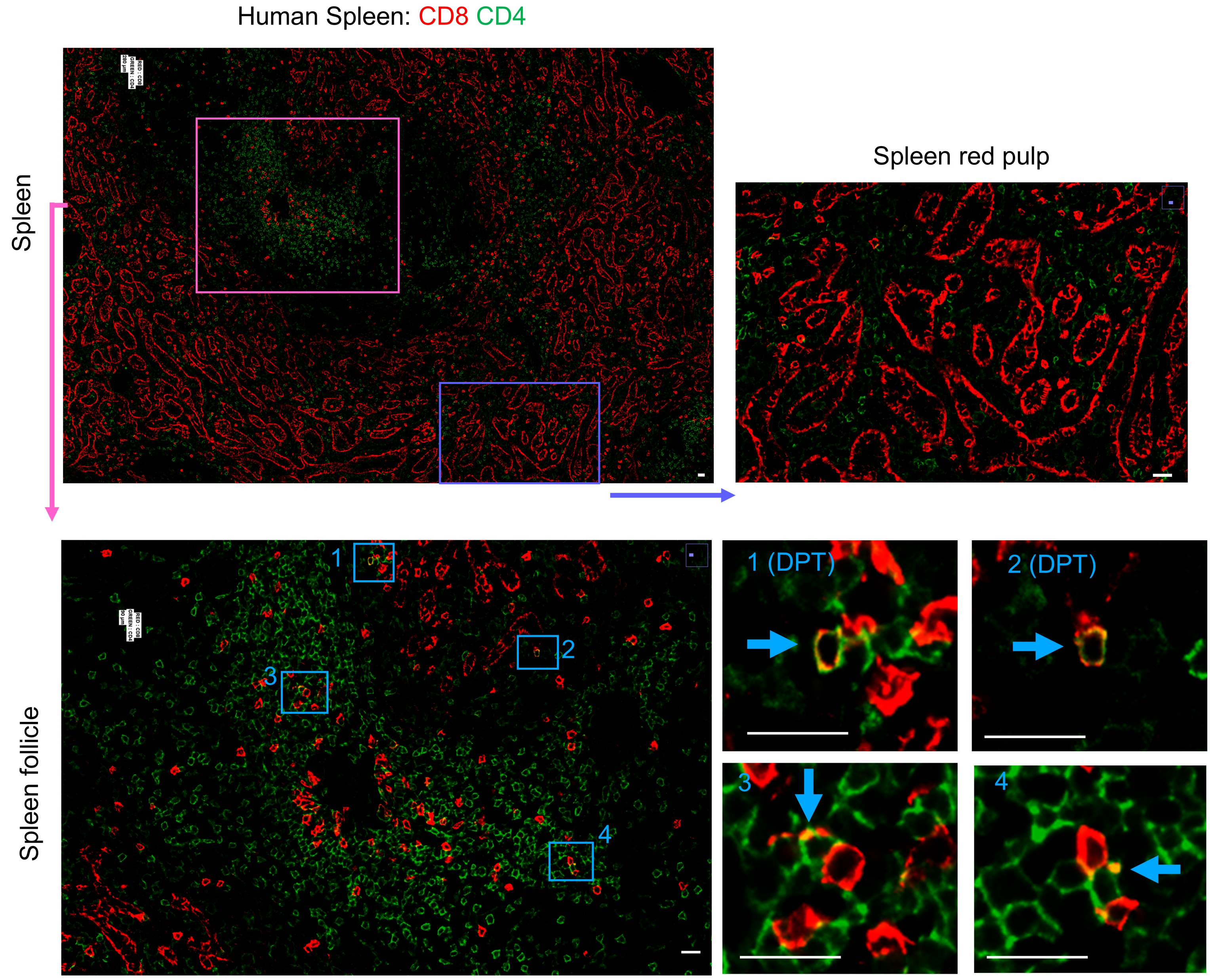

Supplemental Figure 4. CD4 and CD8 immunofluorescence staining of human spleens with DPTs.
Top left panel: a low magnification image of the human spleen with chosen regions of follicle (pink borders) and red pulp (purple borders) stained with CD4 (green) and CD8 (red) antibodies.
Top right panel: zoom-in image of the red pulp insert with no detected DPTs.
Bottom panels: zoom-in image of the follicle insert with 4 highlighted inserts (blue) showing yellow surface of double positive staining of 2 DPTs in inserts 1 and 2 (blue arrows pointed).
Scale bars = 50 μm.

Supplemental Figure 5

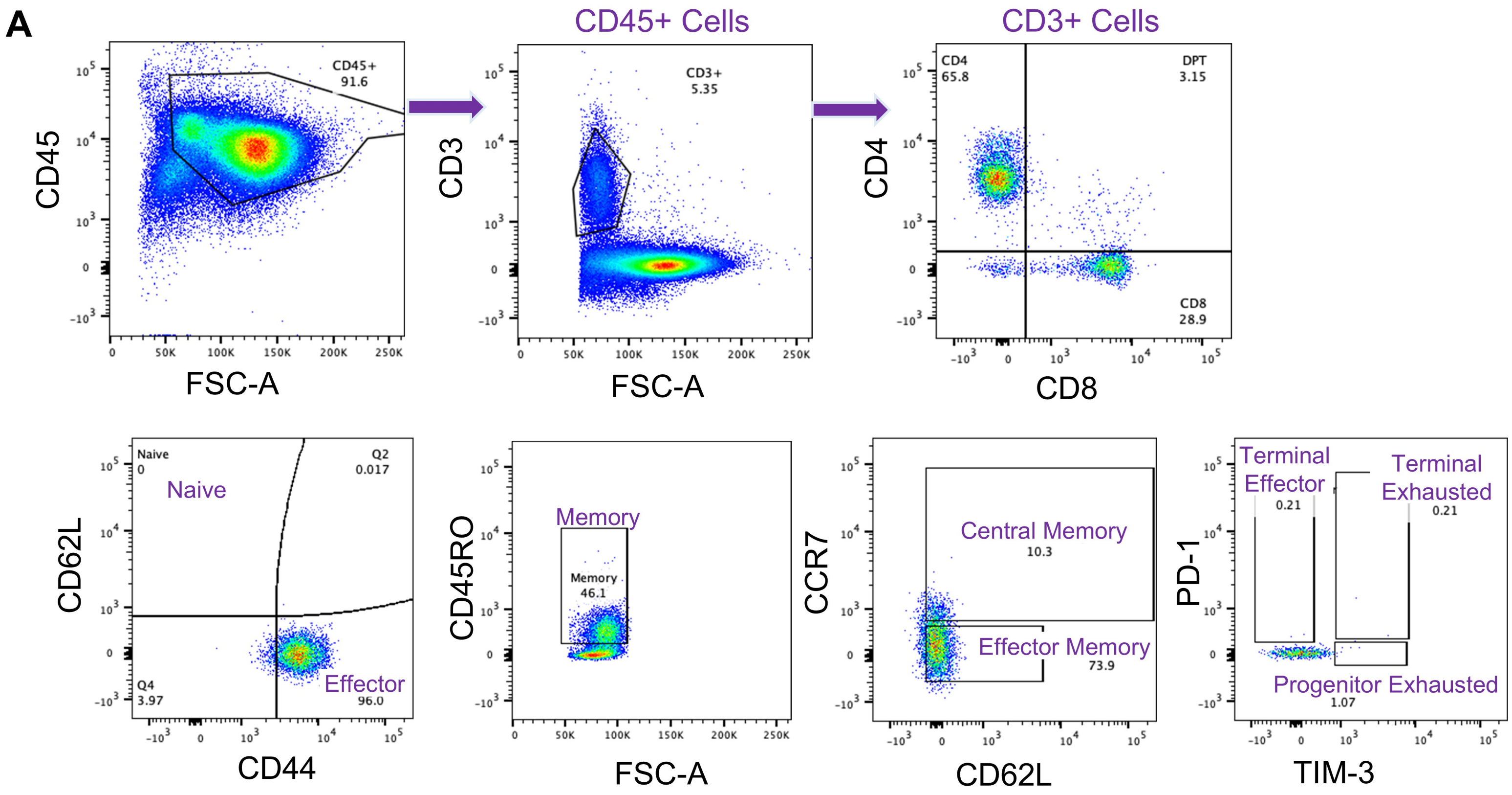

Supplemental Figure 5. Phenotypic characterization of human circulating DPT cells versus $CD4^+$ and $CD8^+$ single positive T cells in WBCs of patients with breast cancer.

A. Representative gating of T/DPT cell subsets, including naïve ($CD62L^+CD44^-$), memory ($CD45RO^+$), central memory ($CD45RO^+CCR7^+$), effector memory($CD45RO^+CCR7^-$), terminal effector ($PD\text{-}1^+TIM3^-$), progenitor exhausted ($TIM3^+PD\text{-}1^-$), terminal exhausted cells ($PD\text{-}1^+TIM3^+$).

Supplemental Figure 6

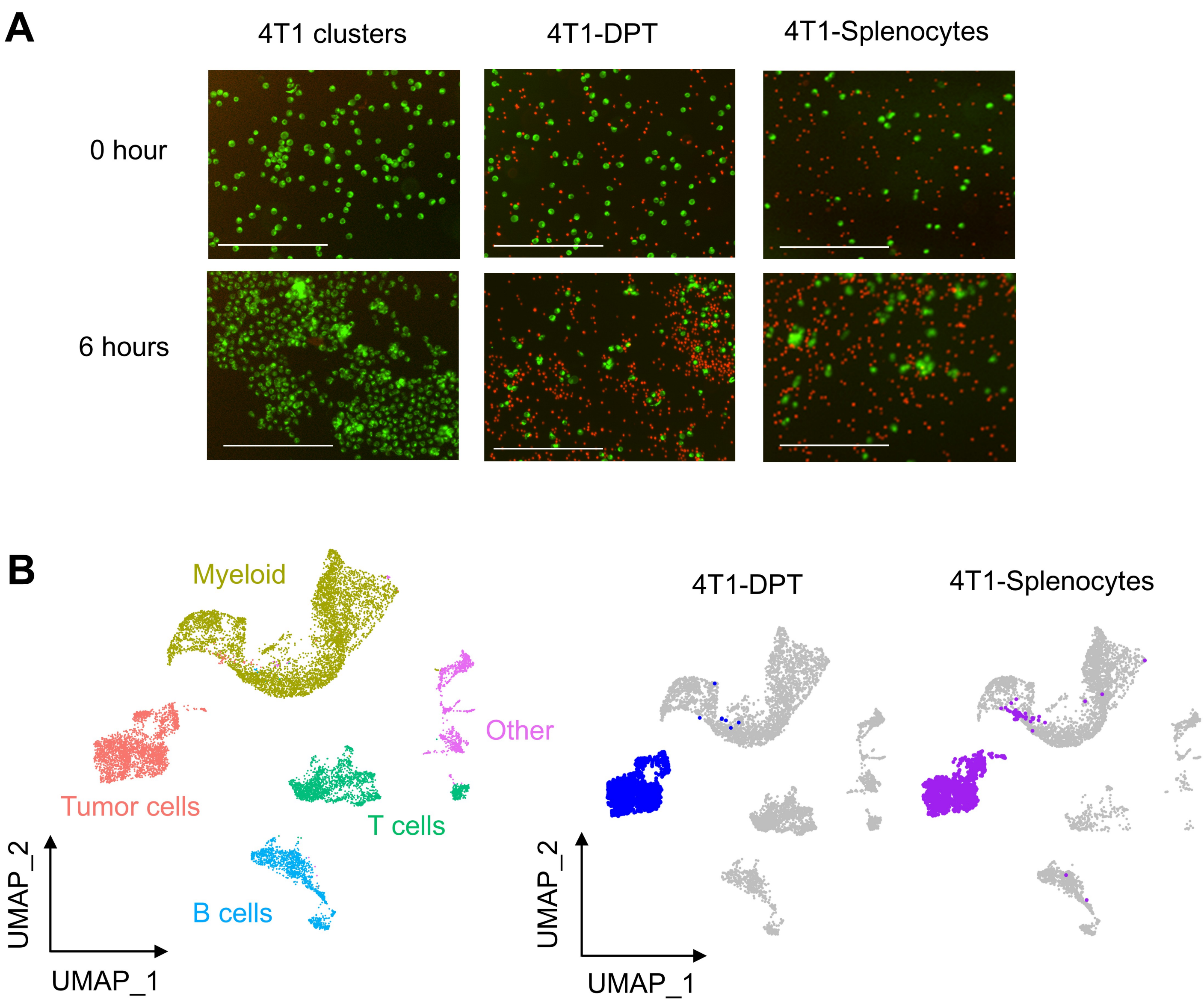

Supplemental Figure 6. 4T1-DPT and 4T1-splenocyte interactions influence tumor cells.

A. Representative images of 4T1 tumor cell only-aggregated homotypic clusters (left panels), 4T1-DPT heterotypic interactions and clusters with DPT cells sorted from tumor-bearing splenocytes (middle panels), and 4T1- splenocytes (unsorted control, right panels) after 6 hours of clustering at 37°C. Green, 4T1 cells labelled by PKH67. Red, DPT cells and splenocytes labeled by Cytolight Red. Scale bars = 300 µm.

B. UMAP of 4T1-DPT cells and 4T1-splenocytes collected after 6 hours incubation (left). UMAP of tumor cells colored by coincubation cell type (right), blue = DPTs and magenta = splenocytes for co-incubation.

Supplemental Figure 7

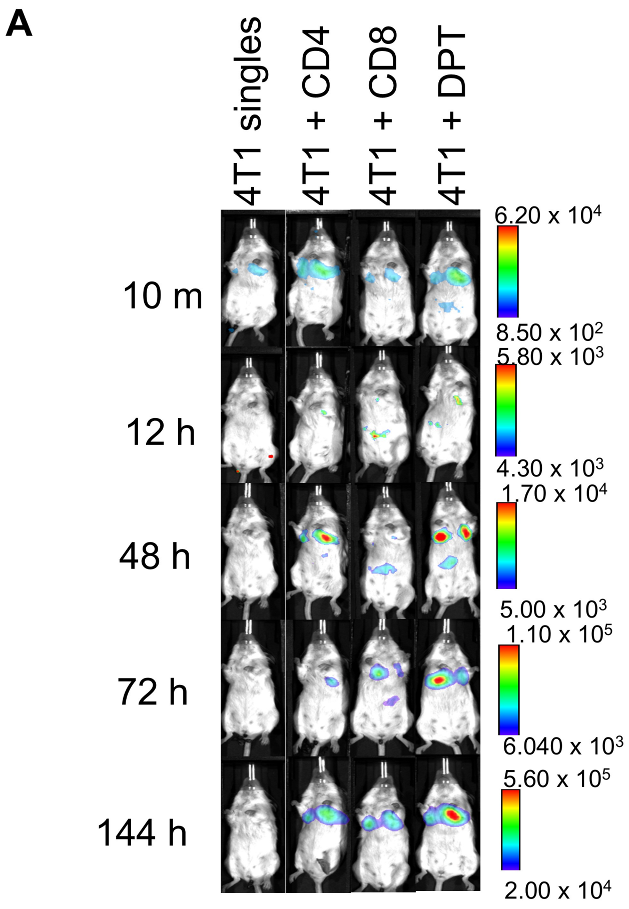

Supplemental Figure 7 Representative bioluminescent images of tail vein colonization.

A. Representative images of *in vivo* bioluminescent signals in mouse lungs of L2T$^+$ 4T1 tumor cells after clustering and tail vein injection,

Supplemental Figure 8

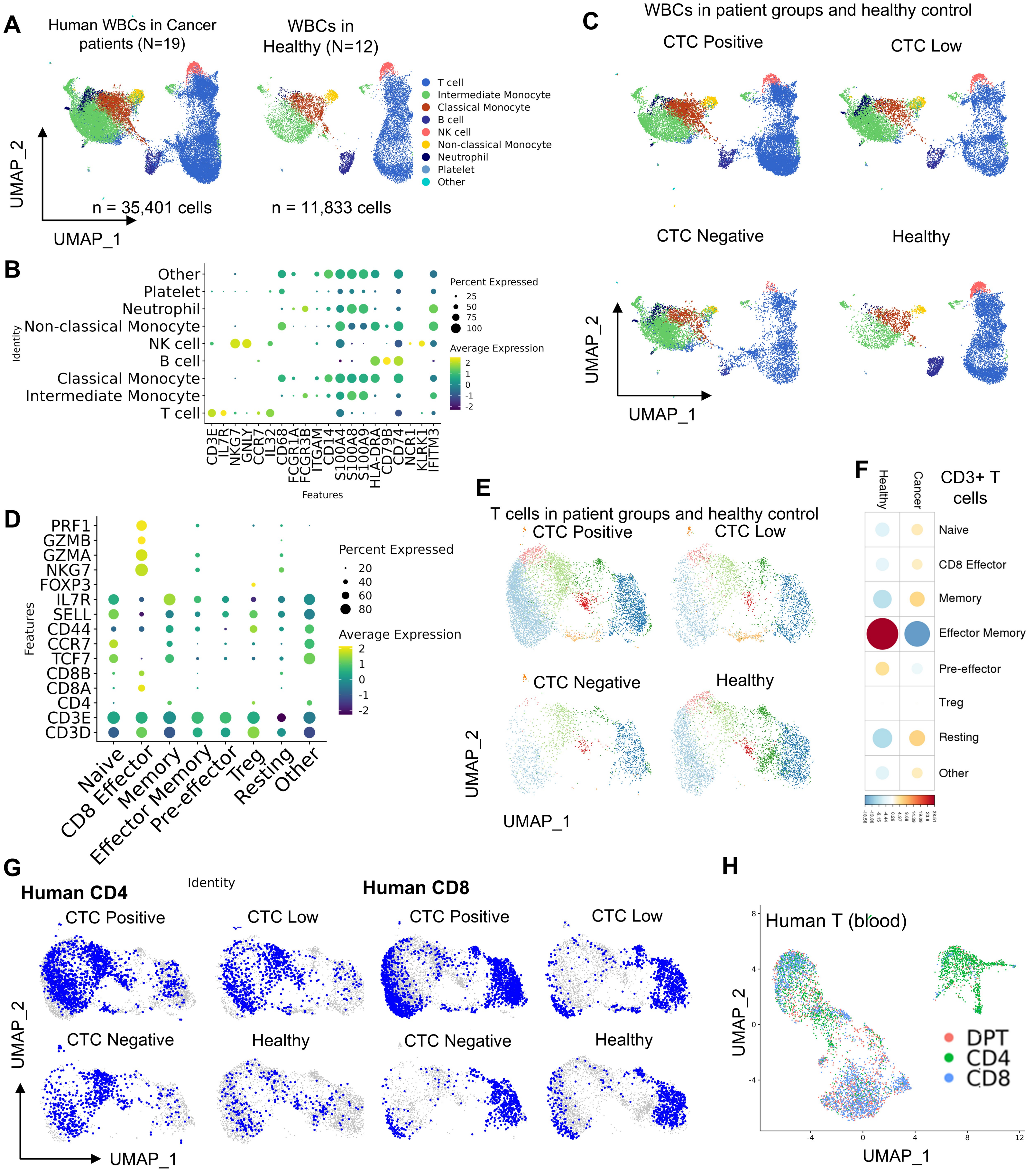

Supplemental Figure 8. scRNA-seq analysis of human WBCs and DPT cells

A. UMAP plots of WBCs collected from breast cancer patients (left panel, N = 19 patients, with 35,401 cells) and healthy controls (right panel, N =12, with n = 11,833 cells). Cell populations are indicated in distinct colors.

B. Dot plot of representative markers used to annotate broad immune cell subsets in human WBCs. Dot size corresponds to the percent of expressing cells, and color corresponds to the expression levels.

C. UMAP plots of WBCs split by CTC status, including breast cancer patients that are CTC positive (≥5 CTCs), CTC low (1-4 CTCs), and CTC negative (0 CTCs) per 7.5 mL blood, and healthy controls (non-cancer).

D. Dot plots depicting representative genes used to annotate T cell subsets

E. Human T cell subset UMAP plots of patient WBCs split by CTC status, including CTC positive, CTC low, and CTC negative, and healthy controls.

F. Correlation plots depicting chi-squared test residuals to determine over- or under-enrichment of each T cell subset from the T cells of cancer patients vs. healthy controls. Dot size corresponds to the absolute value of correlation coefficients, and color corresponds to chi-square residuals

G. CD4 (left) and CD8 (right) T cell distribution in the UMAP plots within four groups as split by CTC status.

H. UMAP plot of combined human circulating T (CD4 and CD8) and DPT cells from our blood cell dataset and a public dataset (*Zhang et al.*), which show partially overlapping profiles between DPT and single positive-cell controls.

Supplemental Figure 9

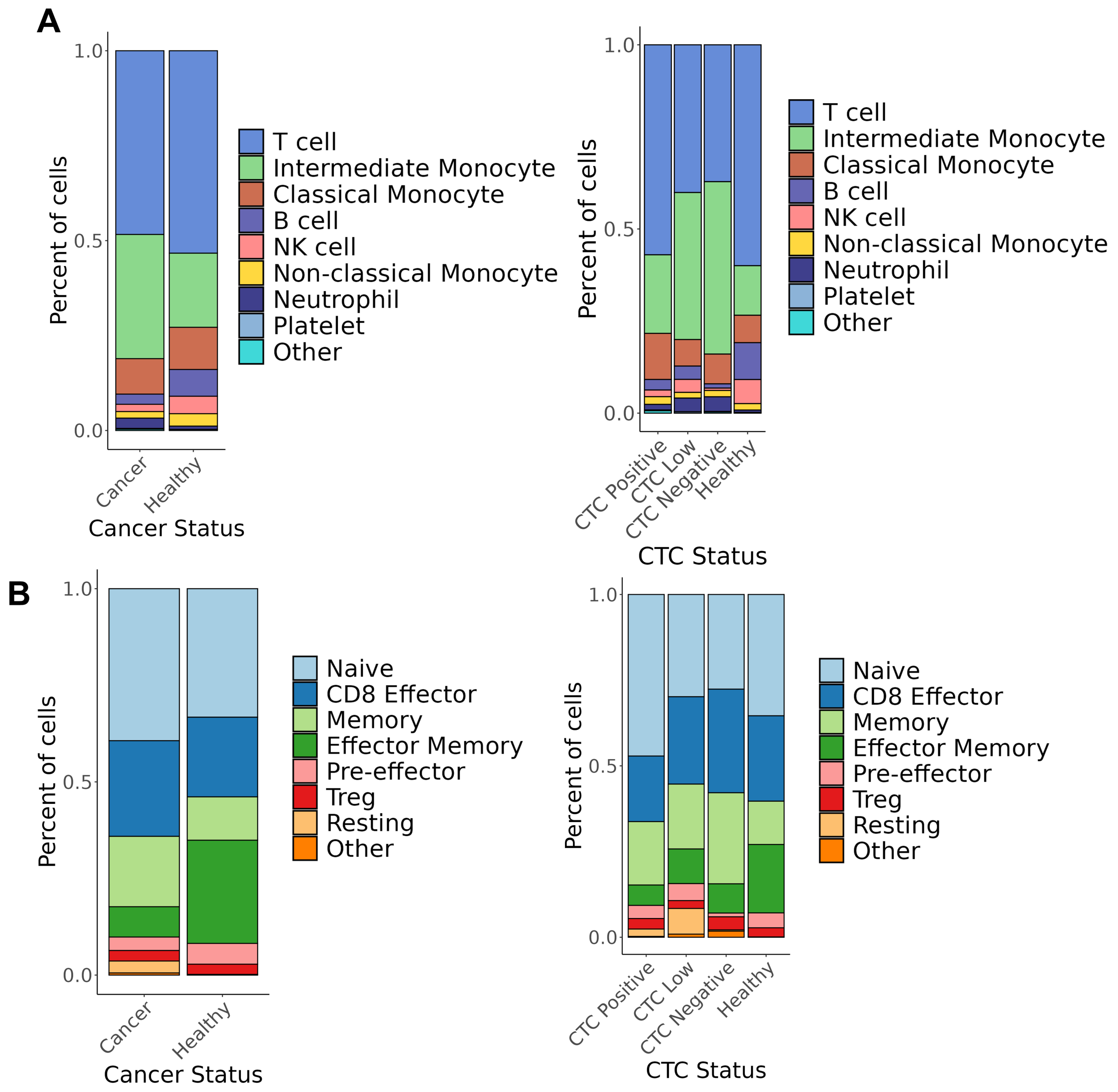

Supplemental Figure 9 Proportions of scRNA-seq immune cell clusters.

A. Bar plot depicting proportions of broad immune cell clusters stratified by cancer status (left) or CTC status (right). **B.** Bar plot depicting proportions of T cell clusters stratified by cancer status (left) or CTC status (right).

Supplemental Figure 10

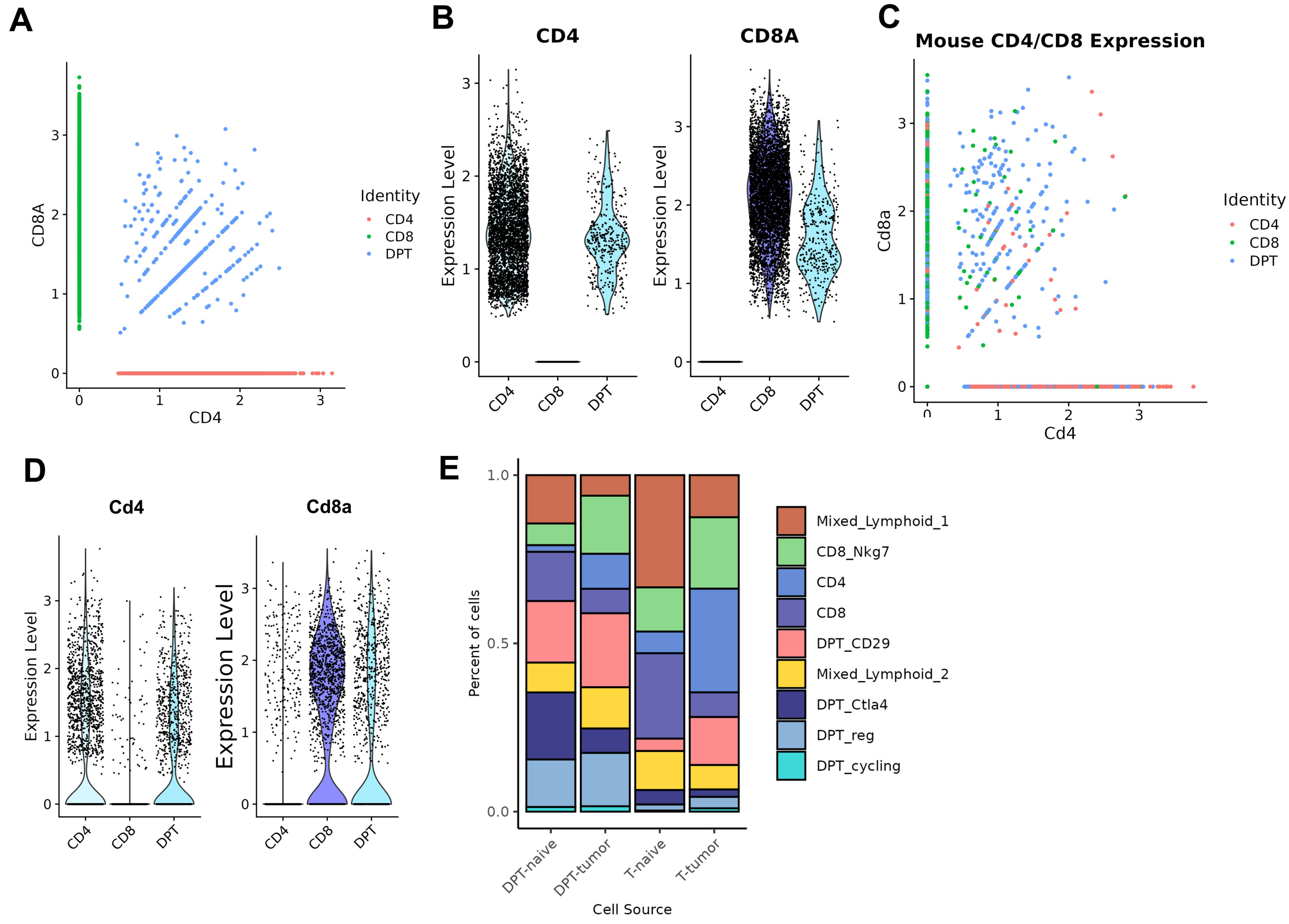

Supplemental Figure 10. Identification of DPT cells in human and mouse scRNA-seq datasets. A-D. Scatterplot (**A, C**) and violin plot (**B, D**) depicting *CD4/Cd4* and *CD8A/Cd8a* expression in human (**A ,B**) and mouse (**C, D**) T cells, separated by T cell identity. **E.** Proportions of DPT and single positive T cells in each mouse scRNA-seq cluster, stratified by source (tumor naïve mouse or tumor bearing mouse) as determined by multiplexing.

Supplemental Figure 11

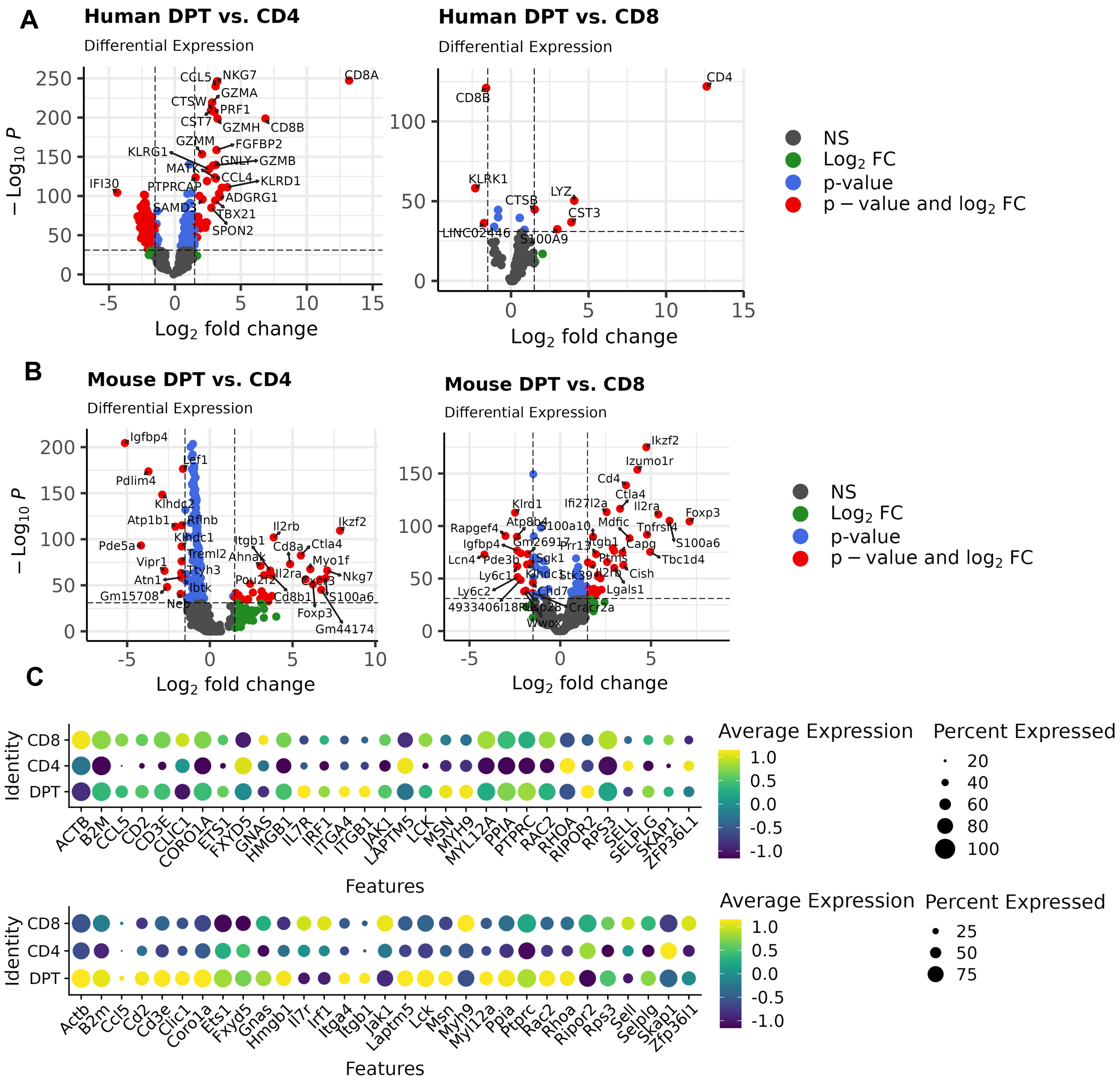

Supplemental Figure 11. Differentially expressed genes between DPT cells and single positive T cells in human and mouse scRNA-seq datasets. A-B. Differential expression of genes between DPT cells and CD4 T cells (left) and CD8 T cells (right) in human (**A**) datasets and mouse (**B**) datasets. **C.** Dot plots depicting normalized expression of candidate adhesion molecules identified in **Figure 3I** in human (top) and mouse (bottom) DPT and single positive T cells.

Supplemental Figure 12

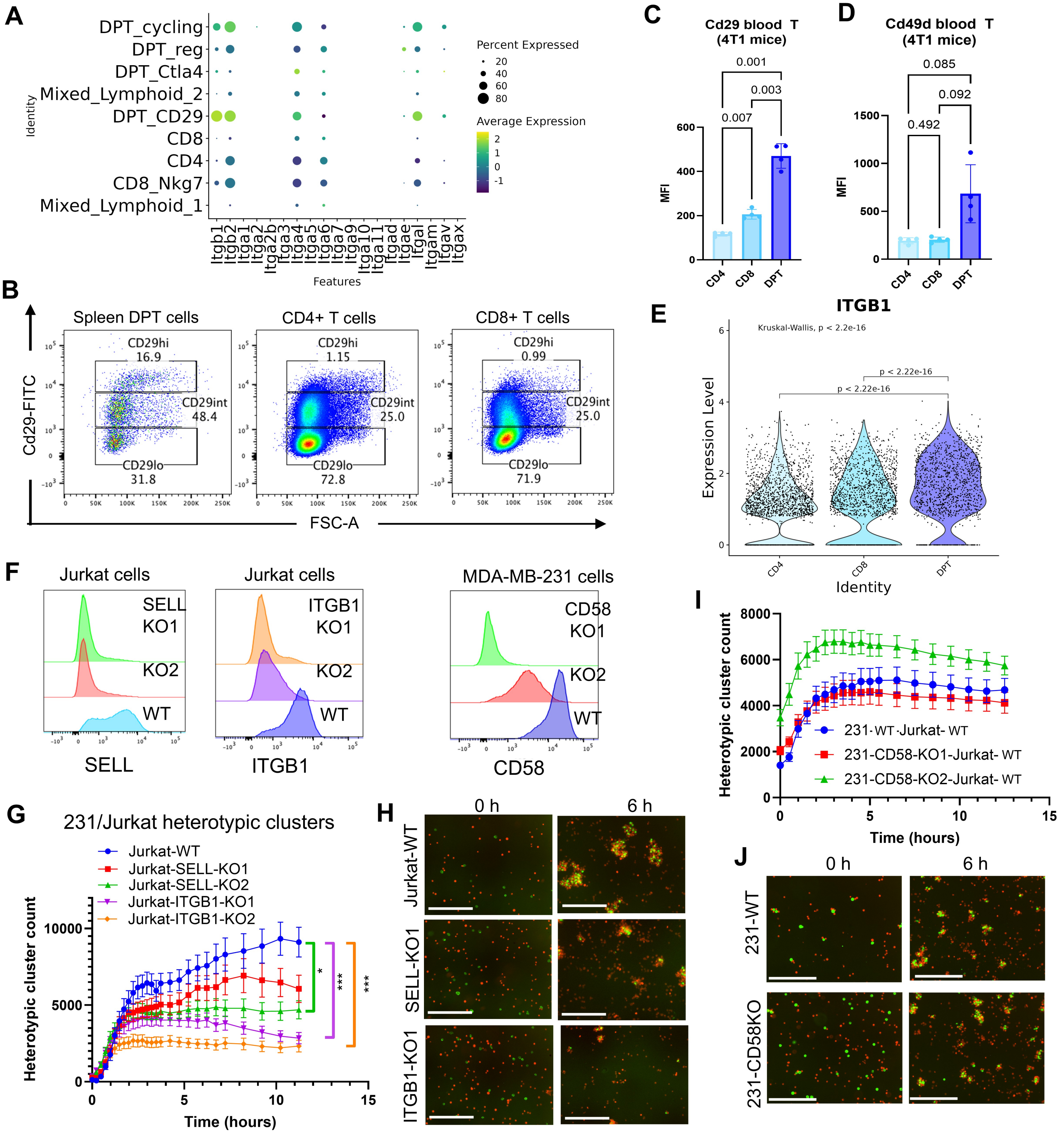

Supplemental Figure 12. Integrins and VCAM1 as mediators of T cell-tumor cell clustering. A. Dot plot of integrin alpha and beta gene expression in mouse splenic T and DPT cells. **B.** Flow plots of Cd29 protein expression in splenic DPT, CD4, and CD8 cells. **C-D.** Bar graphs of Cd29 (C) and Cd49d (D) expression (MFI) in mouse DPT, CD4, and CD8 cells from WBCs. One-way ANOVA P values (N=4 mice). **E.** Violin plots of human ITGB1/CD29 expression in DPT, CD4, and CD8 T cells. **F.** Flow histograms of Jurkat-Cas9-KO cells (SELL and ITGB1) and MDA-MB-231-Cas9-KO cells (CD58). **G.** Curves of heterotypic cluster counts of Jurkat-Cas9 cells, control (NT) or genetic knockout of *SELL* or *ITGB1,* and MDA-MB-231 tumor cells. Graphs represent mean ± standard error of the mean. Two-sided unpaired t-test. *, $p < 0.05$; ***, $p < 0.005$. N = 6 biological replicates. **H.** Representative images of G. **I.** Curves depicting heterotypic interactions between WT Jurkat cells and MDA-MB-231-CD58KO cells. Graphs represent mean ± standard error of the mean. N = 6 biological replicates. **J.** Representative images of the curves from I. Green, MDA-MB-231 cells (231); red, Jurkat cells. Scale bars = 300 µm.

Supplemental Figure 13

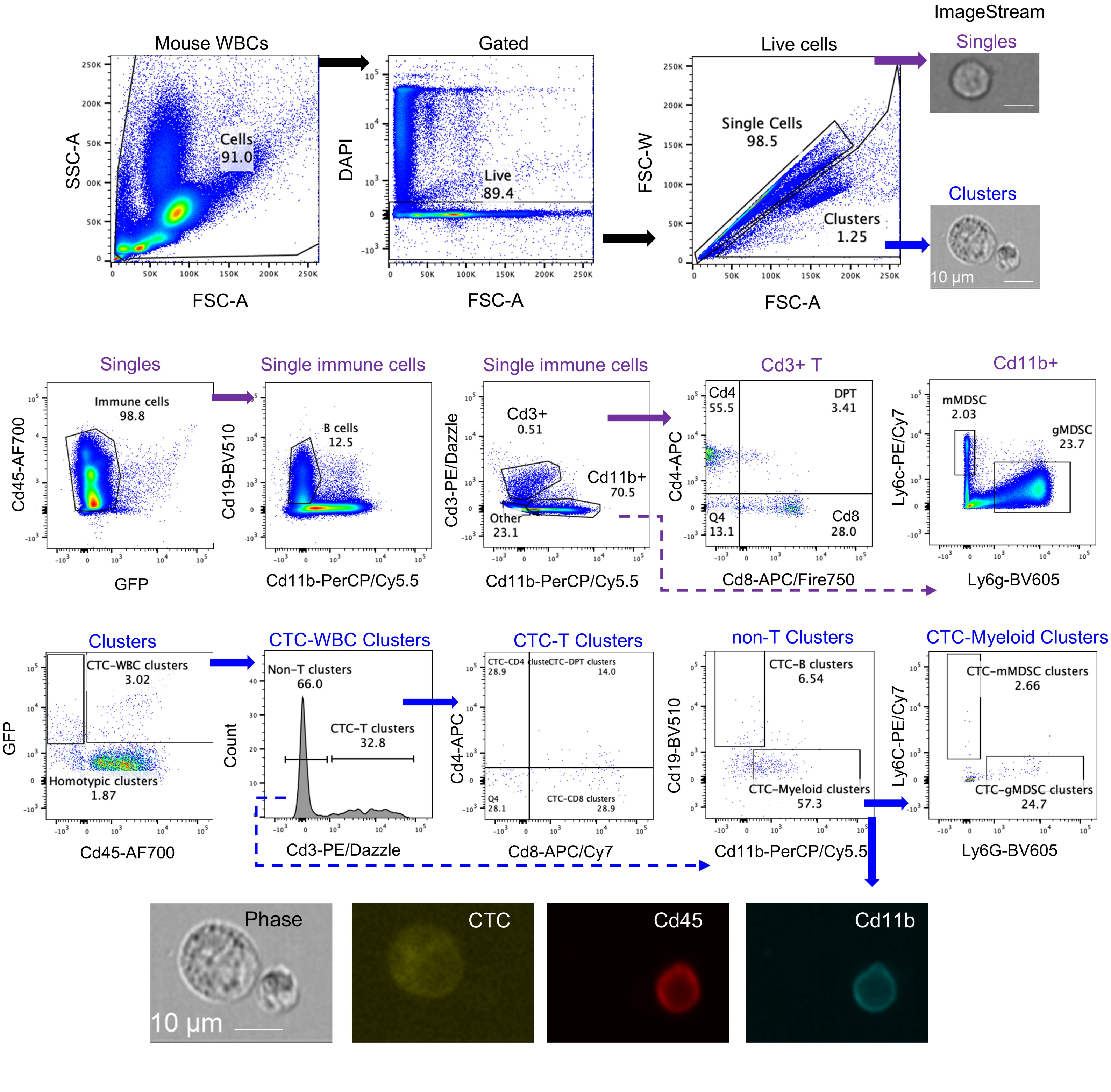

Supplemental Figure 13. Gating strategies for identifying mouse white blood cell (WBC) lineages in singles and CTC-WBC heterotypic clusters. The sequential gates include the top row: mouse WBCs, live cells (DAPI-), singles and clusters. Second row: single cells, single immune cells ($CD45^+$ GFP^-), $Cd3^+$ T cells, $Cd4^+$ and $Cd8^+$ single positive T cells and DPT cells; $Cd19^+$ B cells, Cd $11b^+$ myeloid cells, mMDSC ($Ly6c^+$), and gMDSC ($Ly6g^+$). The third row: clusters, CTC-WBC clusters (eGFP+Cd45+), CTC-T clusters (Cd3+), non-T clusters (Cd3-), CTC-B clusters (CD19+Cd11b-), CTC-myeloid clusters (CD19-CD11b+), CTC-mMDSC clusters (Ly6c+Ly6G-), and CTC-gMDSC clusters (Ly6C-Ly6G+). The bottom row: ImageStream images of CTC-WBC cluster with a CTC and a CD45+CD11b+ WBC in the cluster.

Supplemental Figure 14

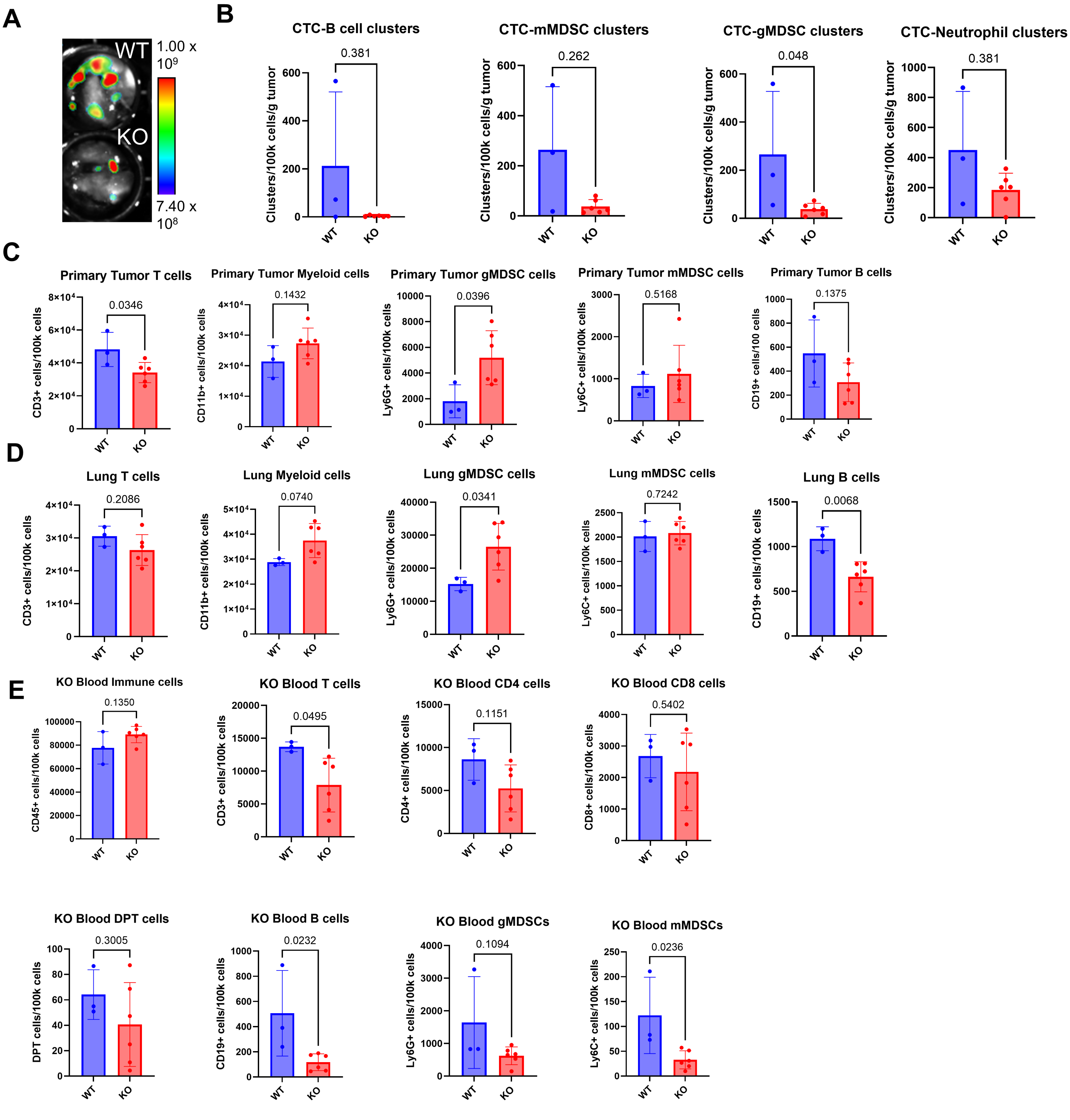

Supplemental Figure 14. Immune cell profiles in heterotypic CTC clusters, primary tumors, lungs, and blood in 4T1-WT/*Vcam1*KO mice.

A. Representative images of *ex vivo* lung biofluorescent signal in mice with 4T1-WT or *Vcam1*KO tumors

B. Bar graphs of various blood heterotypic CTC-WBC clusters, including B cells, mMDSC (CD11b+Ly6C+), gMDSC (CD11b+Ly6G+), and neutrophils (CD11b+Ly6G+Ly6C+) in the blood of 4T1 tumor, WT and Vcam1 KO (KO) mice on Day 9 after orthotopic implantation in Figure 5E.

C-E. Flow cytometry analysis of T cells ($CD3^+$), myeloid cells (CD11b+), gMDSCs (CD11b+Ly6G+), mMDSCs (CD11b+Ly6C+), and B cells (CD19+) in primary tumor (C), lungs (D), and blood (E) of 4T1-WT or 4T1-*Vcam1* KO tumor-bearing mice. Unpaired two-tail t-test, n = 3 for WT control, n = 6 for KO.

Supplemental Figure 15

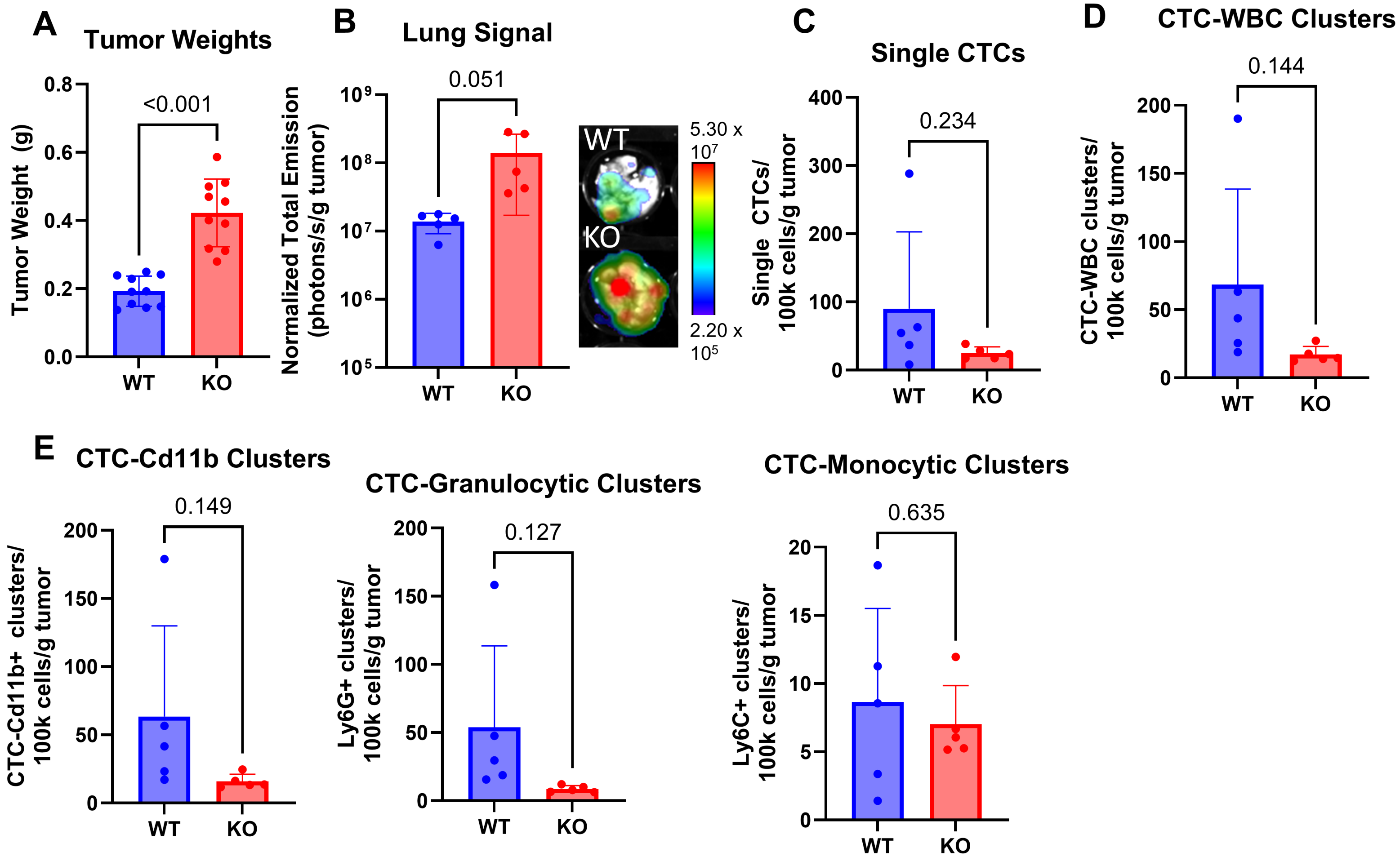

Supplemental Figure 15. Immune cell profiles in heterotypic CTC clusters of NSG mice.

A. Primary tumor volume of 4T1-WT and *Vcam1*KO tumors in NSG mice.

B. Bar graphs of lung bioluminescent signal and representative images of metastatic nodules in 4T1 WT and Vcam1KO tumor bearing NSG mice

C-E. Bar graphs of single CTCs (**C**) and blood heterotypic CTC-WBC clusters (**D**), mMDSC (CD11b+Ly6C+), gMDSC (CD11b+Ly6G+) (**E**) in the blood of 4T1-WT and *Vcam1*KO NSG mice on Day 10 after orthotopic implantation in NSG mice. Two tailed unpaired t-test p values displayed on graphs. N = 5.

Supplemental Figure 16

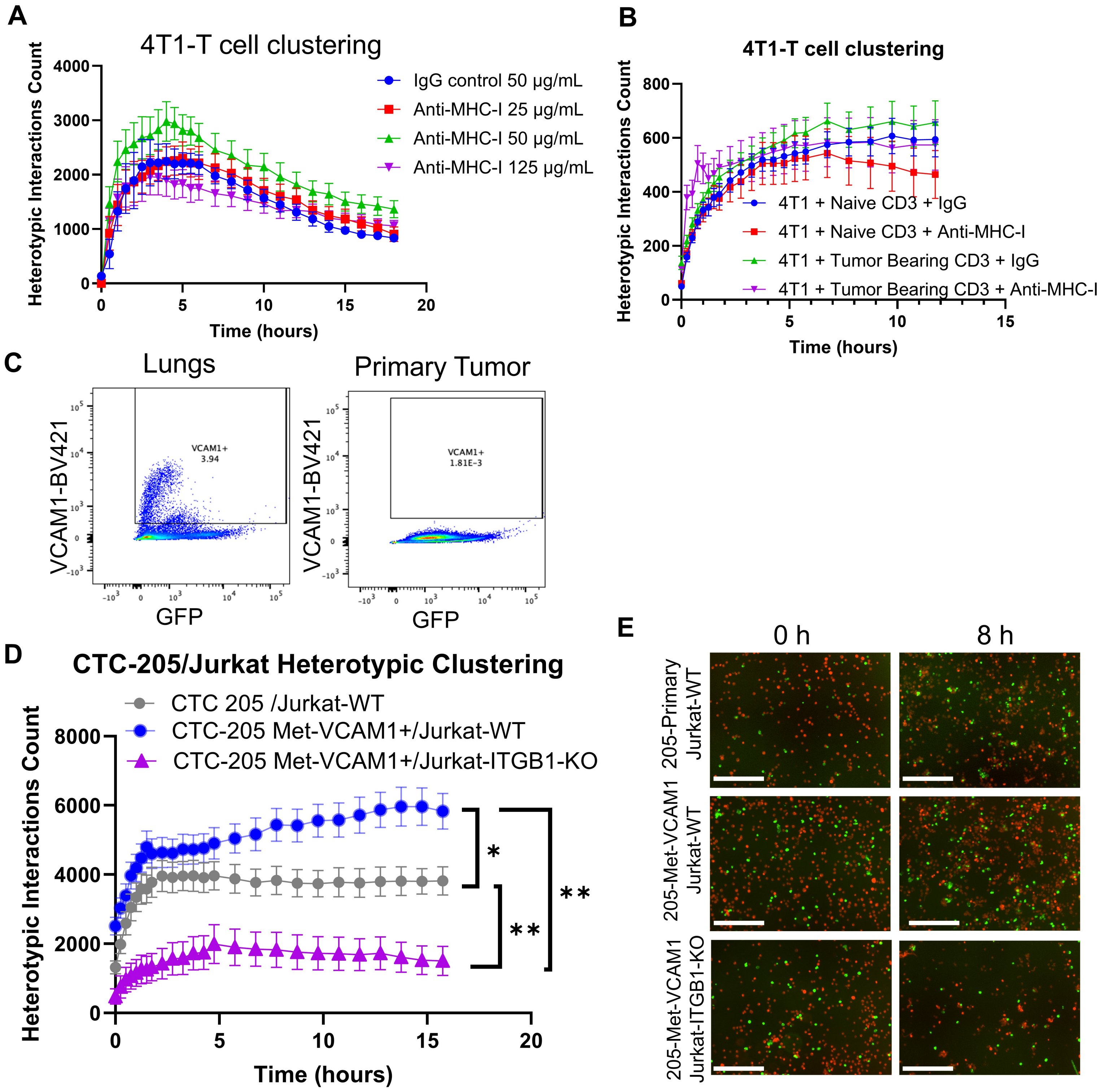

Supplemental Figure 16. ITGB1 and VCAM1 as mediators of T cell-tumor cell clustering.

A. Curves depicting heterotypic interactions between mouse splenic T cells and 4T1 cells with pre-treatment of neutralizing control IgG antibody or MHC-I antibody. Graphs represent mean ± standard error of the mean. N = 6 biological replicates.

B. Curves depicting heterotypic interactions between mouse splenic T cells from 4T1-WT tumor naïve or tumor bearing mice and 4T1 cells with pre-treatment of neutralizing control IgG antibody or MHC-I antibody (50 μg/mL). Graphs represent mean ± standard error of the mean. N = 6 biological replicates.

C. Flow cytometry plots showing VCAM1 expression in PDX-CTC-205 lung metastatic lesions (left) or primary tumor (right).

D-E. Curves (C) and Representative images (D) depicting heterotypic interactions between Jurkat-Cas9-WT or ITGB1KO cells and PDX-CTC-205 primary tumor cells or lung metastatic lesions. Two-way ANOVA, *, $p < 0.05$; **, $p < 0.01$. N = 6 biological replicates. Graphs represent mean ± standard error of the mean. Scale bars = 300 μm.

Supplemental Figure 17

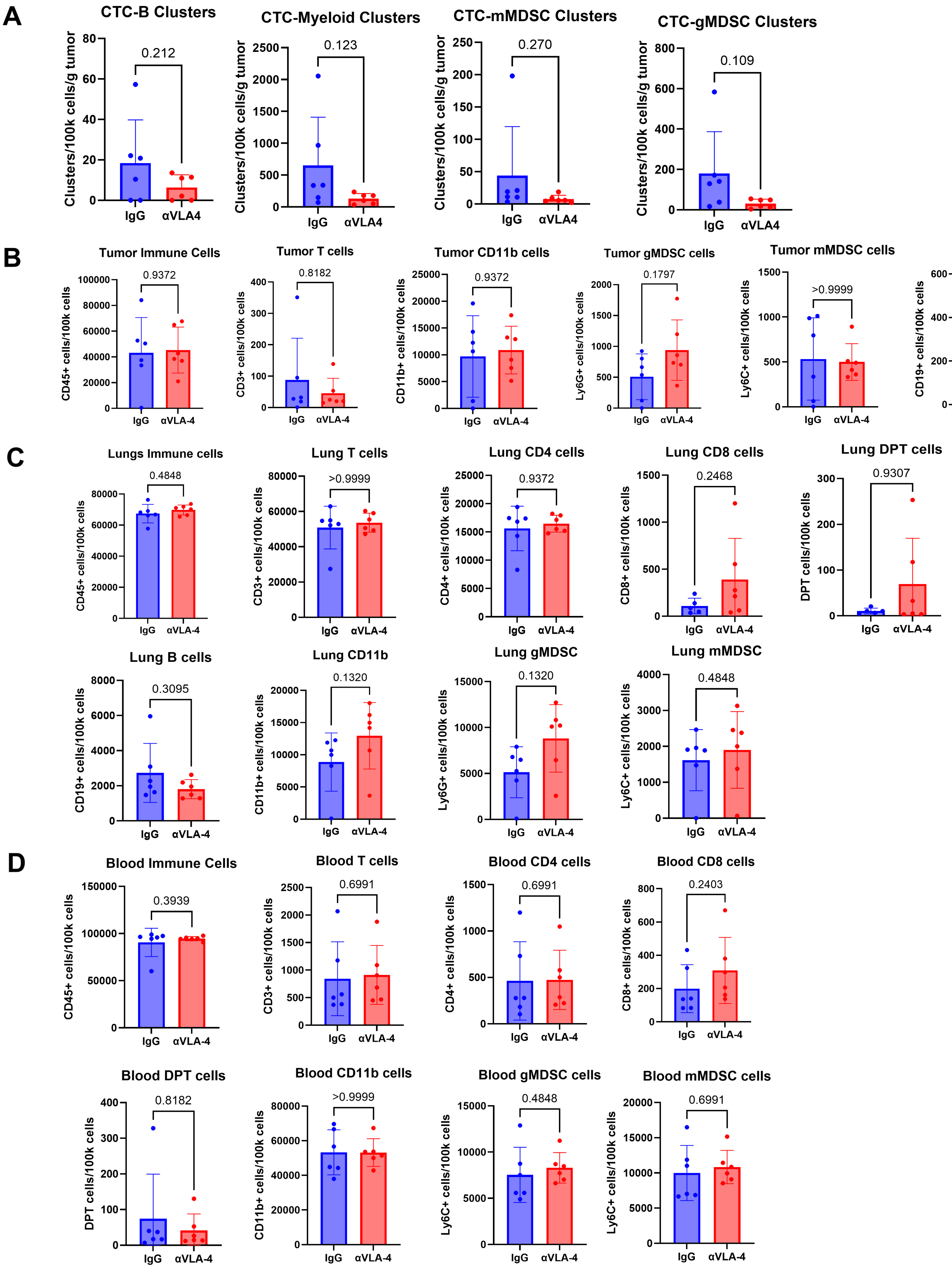

Supplemental Figure 17. Immune cell profiles of primary tumors, lungs, and blood cells from the αVLA4 treated mice and the IgG control (related to Figure 5 I-L).

A-D. Bar graphs of flow cytometry data of immune cells ($CD45^+$), T cells ($CD3^+$), myeloid cells ($CD11b^+$), gMDSCs ($CD11b^+Ly6G^+$), mMDSCs ($CD11b^+Ly6C^+$), and B cells ($CD19^+$) in CTC-WBC clusters (A), primary tumor (B), lungs (C), and blood (D) of IgG or αVLA4 (anti-VLA4) treated mice. Unpaired two-tail t-test, N = 6 mice each group.
